# Structural determinants of the catalytic mechanism of *Plasmodium* CCT, a key enzyme of malaria lipid biosynthesis

**DOI:** 10.1038/s41598-018-29500-9

**Published:** 2018-07-25

**Authors:** Ewelina Guca, Gergely N. Nagy, Fanni Hajdú, Lívia Marton, Richard Izrael, François Hoh, Yinshan Yang, Henri Vial, Beata G. Vértessy, Jean-François Guichou, Rachel Cerdan

**Affiliations:** 1 Dynamique des Interactions Membranaires Normales et Pathologiques, UMR 5235, CNRS, Université de Montpellier, Montpellier, France; 2 Department of Applied Biotechnology and Food Science, Budapest University of Technology and Economics, Budapest, Hungary; 3 Institute of Enzymology, Research Centre for Natural Sciences, Hungarian Academy of Sciences, Budapest, Hungary; 4 Doctoral School of Multidisciplinary Medical Science, University of Szeged, Szeged, Hungary; 5 CNRS UMR5048, Centre de Biochimie Structurale, Université de Montpellier, Montpellier, France; 6 INSERM U1054, Montpellier, France; 7 Present Address: Institute for Research in Biomedicine, The Barcelona Institute of Science and Technology, Carrer de Baldiri Reixac 10, 08028 Barcelona, Spain; 8 Present Address: Division of Structural Biology, University of Oxford, Roosevelt Drive, Oxford, OX37BN United Kingdom

## Abstract

The development of the malaria parasite, *Plasmodium falciparum*, in the human erythrocyte, relies on phospholipid metabolism to fulfil the massive need for membrane biogenesis. Phosphatidylcholine (PC) is the most abundant phospholipid in *Plasmodium* membranes. PC biosynthesis is mainly ensured by the *de novo* Kennedy pathway that is considered as an antimalarial drug target. The CTP:phosphocholine cytidylyltransferase (CCT) catalyses the rate-limiting step of the Kennedy pathway. Here we report a series of structural snapshots of the *Pf*CCT catalytic domain in its free, substrate- and product-complexed states that demonstrate the conformational changes during the catalytic mechanism. Structural data show the ligand-dependent conformational variations of a flexible lysine. Combined kinetic and ligand-binding analyses confirm the catalytic roles of this lysine and of two threonine residues of the helix αE. Finally, we assessed the variations in active site residues between *Plasmodium* and mammalian CCT which could be exploited for future antimalarial drug design.

## Introduction

Malaria is a major global health problem and the most life-threatening parasitic disease^1^. In 2016, 216 million cases of malaria and 445 000 deaths were estimated^2^. *Plasmodium falciparum* is the parasite that causes the most fatal form of malaria among all species infecting humans. While the widespread antimalarial efforts featured by the use of insecticide-impregnated bed nets and the artemisinin-based combination therapies (ACT) yielded a marked decrease in malaria incidence until 2015, the rate of decline has recently stalled^2^. Further progress in reducing malaria may be compromised due to the emergence of artemisinin resistant parasites^3,4^. Therefore, it is necessary to search alternative antimalarial therapeutics involving novel targets and mechanisms of action.

**Figure 1:**
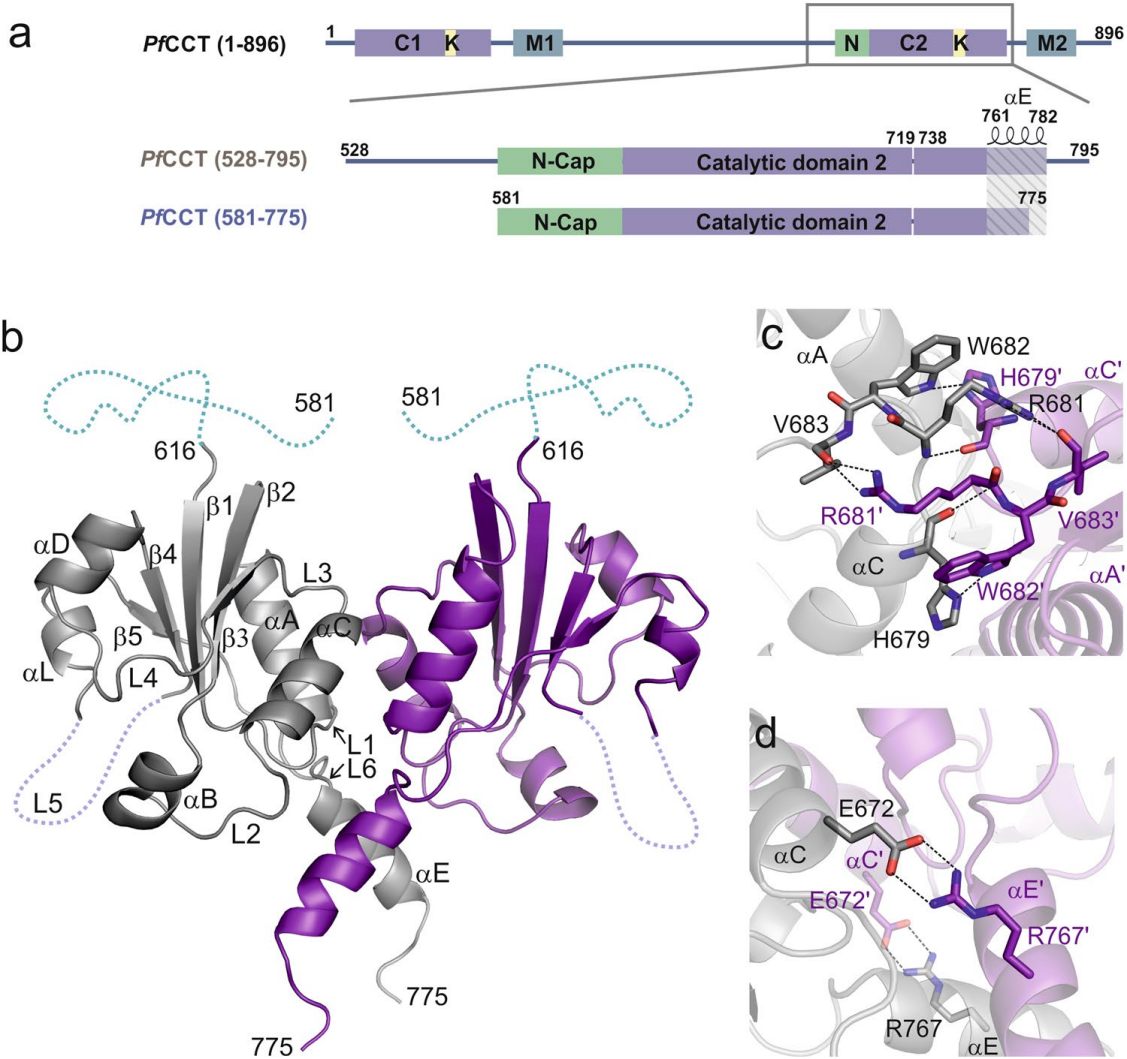
*Pf*CCT domain organization and structure of *Pf*CCT_(581–775)_. (**a**) Domain organization of the full length *Pf*CCT, *Pf*CCT_(528–795)_ and *Pf*CCT_(581–775)_ catalytic domain constructs, lacking a lysine-rich loop between residues 720–737. N-Cap segment (N), Catalytic domains (C), membrane binding domains (M) and lysine-rich sequences (K) are indicated. (**b**) Cartoon representation of *Pf*CCT_(581–775)_ dimer (PDB:4ZCT). Nomenclature of secondary structures follows that of *Rn*CCT^20^ and *B. subtilis* GCT^34,36^ in this order β1-L1-αA-β2-αB-L2-αC-L3-β3-L4-αD-β4-L5-αL-β5-L6-αE. The N-terminal disordered part assigned by NMR is depicted as blue dashed line (see also Supplementary Fig. S2). The flexible loop L5 lacking a lineage-specific lysine-rich region (720–737) is indicated by a violet dashed line. (**c**,**d**) Close-up of dimer interface regions. Residues involved in inter-monomer contacts (dashed line) are shown as sticks. Primes indicate residues and secondary structures of the other monomer.

During the symptomatic erythrocytic stage of *P. falciparum*, the parasite relies on phospholipids to build the membranes necessary for intense cell division. The major phospholipid is phosphatidylcholine (PC) accounting for 40–50% of the total phospholipid content and the parasite possesses its own enzymatic machinery to produce PC^5,6^. The *de novo* Kennedy pathway is the main route for the PC synthesis in *P. falciparum* and has been identified as a pharmacological target for the treatment of malaria^7–10^. Recently, a link has been established between the level of lysophosphatidylcholine, a major supplier of choline for the Kennedy pathway, and the sexual stage differentiation in *P. falciparum*^11^. This emphasizes the importance of phospholipid biosynthesis at different stages of the parasite development. The second step of the PC biosynthesis process catalyzed by CTP:phosphocholine cytidylyltransferase (CCT) [EC: 2.7.7.15] is rate limiting and essential for the survival of the murine parasite at its blood stage^12,13^. Partial inhibition of *Pf*CCT is also part of the mechanism of action of antimalarial compounds targeting lipid biosynthetic pathways^14,15^. CCT transfers a cytidylyl group from CTP to phosphocholine (ChoP) via a mechanism involving the sequential binding of both substrates to form a ternary complex^16–19^. This leads to the formation of CDP-choline (CDPCho) by the nucleophilic attack of the ChoP phosphomonoester on the α-phosphate of CTP and the release of pyrophosphate (PP_i_) as an additional product^19,20^. CCTs are amphitropic enzymes that display a several-fold activity increase upon binding to phosphatidylcholine depleted membrane surfaces by relieving an auto-inhibitory contact between its catalytic and membrane binding domains^21,22^. Most eukaryotic CCTs contain a single catalytic and membrane binding domain and form dimers^22^. In contrast, the gene of *Pf*CCT underwent a domain duplication event and thus encodes two catalytic and two membrane binding domains connected by a disordered interdomain segment^16,23^ (Fig. 1a). The N-terminal (C1) and C-terminal (C2) catalytic domains share 91% sequence identity over 180 amino-acids. To date, only two high resolution crystal structures of *Rattus norvegicus* CCT (*Rn*CCT) catalytic domain were determined, both in complex with CDPCho^20,24^. The first *Rn*CCT crystal structure showed an α/β Rossmann fold for the catalytic domain conserved in cytidylyltransferase enzymes and allowed the identification of key enzyme interactions with CDPCho^20^. The second crystal structure of the *Rn*CCT catalytic domain was solved in the presence of the amphipathic auto-inhibitory (AI) helix of the membrane binding domain^24^. These structural and additional biochemical studies^25,26^ provided evidence that the regulatory mechanism of CCT involves a direct interaction between the C-terminal helix αE of the catalytic domain and the AI helix. Many aspects of the CCT structure and regulatory mechanism have been uncovered recently^21,22,27^. However, 3D structures of the free and substrate-bound enzyme were still to be determined for the detailed understanding of the catalytic mechanism of CCT.

Here, we present the crystal structures of the catalytic domain of the *P. falciparum* enzyme (*Pf*CCT) in the free form, as well as in complex with its substrate ChoP or fragments (CMP and choline) and with the product CDPCho, thereby visualizing the free and the ChoP-complexed states of CCT for the first time. The set of high resolution structures allowed the identification of active site conformational changes along the catalytic reaction. Structural insights complemented with kinetic and ligand binding analyses elucidate the role of key active site residues in the *Pf*CCT mechanism of action. The presented results allow detailed structural characterization of a potential antimalarial target of the phospholipid metabolism pathway of *Plasmodium* and may help for the design of specific inhibitors.

## Results

### Biochemical characterization of *Pf*CCT catalytic domain construct

In order to provide structural insights into the mechanism of action of *Pf*CCT, we set out to crystallize the constitutively active C-terminal catalytic domain of *Pf*CCT. Several constructs were tested and our attempts were successful only in case of *Pf*CCT_(581–775)_ that corresponds to the exact region visible in the crystal structure of *Rn*CCT (PDB: 3HL4)^20^ (Fig. 1a). While this construct proved to be highly crystallogenic, activity assays revealed its reduced catalytic rate together with an increased K_M_ for CTP as compared to the longer construct *Pf*CCT_(528–795)_ used in our previous studies^16^ (Table 1 and Supplementary Fig. S1). We further assessed the functionality of *Pf*CCT_(581–775)_ by characterizing its substrate and product binding ability. Its affinity for CDPCho (K_d_ = 47 µM) reported by isothermal titration calorimetry (ITC) displayed a moderate attenuation as compared to the one of *Pf*CCT_(528–795)_ (K_d_ = 34 µM) (Table 2 and Supplementary Fig. S1). Saturation Transfer Difference Nuclear Magnetic Resonance (STD-NMR) experiments indicated STD signal of CTP, ChoP and CDPCho bound to *Pf*CCT_(581–775)_ at 1 mM, 4 mM and 1 mM concentrations, respectively (Supplementary Fig. S1). This latter result is consistent with previously observed unusually low ChoP affinity of *Pf*CCT^16^. We concluded that *Pf*CCT_(581–775)_ is compatible with substrate and product recognition and may be amenable to structural studies provided that its compromised enzymatic function is taken into account in structure-based interpretations. It is likely that the drop of *Pf*CCT_(581–775)_ enzyme activity is due to the elimination of the C-terminal flanking part as similarly located truncations in the *Rn*CCT catalytic domain also led to severe loss of activity^28,29^. After unsuccessful efforts to crystallize longer *Pf*CCT catalytic domain constructs with an extended C-terminal end, we continued crystallization trials with *Pf*CCT_(581–775)_ and obtained single crystals of the free enzyme and in presence of the enzymatically relevant ligands ChoP, CMP and CDPCho as well as Cho.

**Table 1:** Kinetic parameters of *Pf*CCT enzyme constructs.

*PfCCT*	*k*_*cat*_ (s^−1^)	*K*_*M,CTP*_ (µM)	*K*_*M,ChoP*_ (µM)
581–775	0.010 ± 0.001	1,100 ± 200	N.D.
528–795^a^	1.45 ± 0.05	170 ± 20	1,950 ± 100
528–795 (K663A)^b^	0.0008 ± 0.0002	70 ± 20	N.D.
528–795 (Y626F/Q636A)	0.24 ± 0.01	170 ± 10	1,600 ± 100
528–795 (T761A)	0.0010 ± 0.0001	1,270 ± 90	3,700 ± 400
528–795 (T762A)	0.0010 ± 0.0001	610 ± 70	2,100 ± 200

^a^Experimental data are from Nagy e*t al*.^16^.

^b^The kinetic constant of *Pf*CCT_(528–795)_ K663A refer to a phosphohydrolase activity (see Supplementary Fig. S4).

Values are the mean (±SD) of at least 2 independent experiments.

N.D.: not determined.

**Table 2:** Thermodynamic parameters of ligand binding to *Pf*CCT enzyme constructs.

*Pf*CCT	K_d,CDP-Cho_ (µM)	ΔH_CDP-Cho_ (kcal/mol)	−TΔS_CDP-Cho_ (kcal/mol)	ΔG_CDP-Cho_ (kcal/mol)	K_d,CTP_ (µM)	ΔH_CTP_ (kcal/mol)	−TΔS_CTP_ (kcal/mol)	ΔG_CTP_ (kcal/mol)
581–775	47 ± 5	−10.5 ± 0.3	4.7 ± 0.4	−5.8 ± 0.1	N.D.	N.D.	N.D.	N.D.
528–795	34 ± 1	−16.1 ± 0.1	10.1 ± 0.1	−6.0 ± 0.2	60 ± 2	−8.6 ± 0.3	3.0 ± 0.4	−5.7 ± 0.1
528–795 (K663A)	210 ± 60	−11.4 ± 1.5	6.5 ± 1.6	−5.0 ± 1.4	520 ± 50	6.1 ± 9.3	−10.5 ± 0.6	−4.4 ± 0.1
528–795 (Y626F/Q636A)	22.2 ± 0.2	−12.0 ± 0.2	5.8 ± 0.1	−6.2 ± 0.1	60 ± 2	−10.8 ± 0.1	5.1 ± 0.2	−5.7 ± 0.1
528–795 (T761A)	N.D.	N.D.	N.D.	N.D.	170 ± 35	−7.0 ± 0.8	1.9 ± 1.0	−5.1 ± 0.1
528–795 (T762A)	N.D.	N.D.	N.D.	N.D.	170 ± 12	−10.5 ± 0.7	5.5 ± 0.8	−5.1 ± 0.1

Values are the mean (±SD) of at least 2 independent experiments, except for *Pf*CCT_(528–795)_ Y626F/Q636A (see Methods).

N.D.: not determined.

### Structure of the free *Pf*CCT C-terminal catalytic domain

The structure of *Pf*CCT_(581–775)_ was determined at 2.2 Å resolution by molecular replacement using *Rn*CCT structure^20^ (PDB: 3HL4) as a search model (Table 3). The enzyme forms a globular dimer with an α/β Rossmann fold that is well conserved in CCTs and other members of the cytidylyltransferase enzyme family (Fig. 1b). Here, the homodimer state likely mimics the full-length *Pf*CCT conformation with two almost identical catalytic sites. The dimerization interface of *Pf*CCT is mediated by inter-molecular contacts between several structural elements of each chain. Residues of helix αC of one chain are in contact with residues of loop L3′, helix αA’ and helix αE’ of the other chain (Fig. 1c,d). In addition, residues of helix αE contact residues of helix αE’ and loop L2′. Intermolecular contacts between L3 loop residues of both chains include the ^681^RWVDE^685^ dimerization motif that is conserved in all cytidylyltransferases. We observed a lack of density in loop L5 for nine residues (^712^IPYANNQK^719^E^738^) in a region where a lysine-rich insertion (720–737) had been removed from the construct^16^. The 35 N-terminal amino acids of *Pf*CCT_(581–775)_ were also not visible on electron density maps. This part corresponds to the N-terminal pre-catalytic region of *Rn*CCT containing two short helices that constitute an ‘N-cap’ by folding on the subsequent conserved catalytic core of the enzyme^20,24^ (Fig. 1a). To obtain structural information on this N-terminal region, we performed ^1^H-^1^H 2D NMR experiments on *Pf*CCT_(581–775)_. The resonances of 33 N-terminal residues were clearly detectable and could be assigned on the ^1^H-^1^H 2D NOESY spectrum (Supplementary Fig. S2), arguing for their likely disorder. Few α-helix specific crosspeaks were found for the ^604^DSEKNKG^610^ segment (Supplementary Fig. S2).

**Table 3:** Crystallographic data and refinement statistics.

	*Pf*CCT (PDB: 4ZCT)	*Pf*CCT-CMP (PDB: 4ZCP)	*Pf*CCT-Cho (PDB: 4ZCQ)	*Pf*CCT-ChoP (PDB: 4ZCR)	*Pf*CCT-CDPCho (PDB: 4ZCS)
**Data collection**					
Space group	I222	I222	I222	I222	I222
Cell dimensions					
*a*, *b*, *c* (Å)	48.5, 74.4, 119.0	50.5, 69.3, 116.4	50.6, 69.4, 119.0	50.6, 69.6, 117.9	115.5,149.8, 176.6
*α*, *β*, *γ* (°)	90, 90, 90	90, 90, 90	90, 90, 90	90, 90, 90	90, 90, 90
Resolution (Å)^a^	63.02–2.22 (2.44–2.22)	58.22–1.98 (2.03–1.98)	59.92–1.92 (1.99–1.92)	46.52–1.80 (1.85–1.80)	113.7–2.45 (2.51–2.45)
*R*_sym_ or *R*_merge_	0.087 (0.497)	0.059 (0.307)	0.038 (0.511)	0.054 (0.497)	0.032 (0.377)
*I*/σ*I*	10.7 (3.0)	10.5 (2.8)	15.0 (1.8)	14.3 (1.8)	10.1 (2.5)
CC1/2	0.995 (0.879)	0.999 (0.896)	0.999 (0.737)	0.999 (0.718)	0.997 (0.859)
Completeness (%)	97.9 (97.9)	99.7 (99.7)	99.2 (99.2)	99.6 (99.6)	99.9 (99.6)
Redundancy	4.0 (3.9)	4.6 (4.6)	4.0 (4.0)	6.4 (6.4)	9.3 (9.4)
B-Wilson	17.84	34.40	44.29	39.00	40.80
**Refinement**					
Resolution (Å)	2.22	1.98	1.92	1.80	2.45
No. reflections	10780	13840	16280	18719	52521
*R*_work_/*R*_free_	20.21/24.26	20.92/24.71	20.40/22.60	17.89/23.22	18.06/23.41
No. atoms					
Protein	1120	1023	1042	1050	6810
Ligands		21	7	11	186
PEG					38
Water	98	43	63	91	347
*B*-factors					
Protein	24.3	41.6	50.4	47.1	45.0
Ligands		38.9	69.3	62.4^b^	34.1
PEG					77.1
Water	28.3	43.7	51.2	53.9	40.8
R.m.s. deviations					
Bond lengths (Å)	0.007	0.008	0.008	0.009	0.018
Bond angles (°)	1.003	1.104	0.965	1.218	1.970
Ramachandran plot					
Most favored regions (%)	97.58	96.80	98.41	99.18	96.37
Allowed regions (%)	2.42	3.20	1.59	0.82	2.91

^a^Values in parentheses are for the highest resolution shell.

^b^The occupancy of ChoP is 50%.

### Co-crystal structures of CMP-, Cho-, ChoP- and CDPCho-bound *Pf*CCT

**Figure 2:**
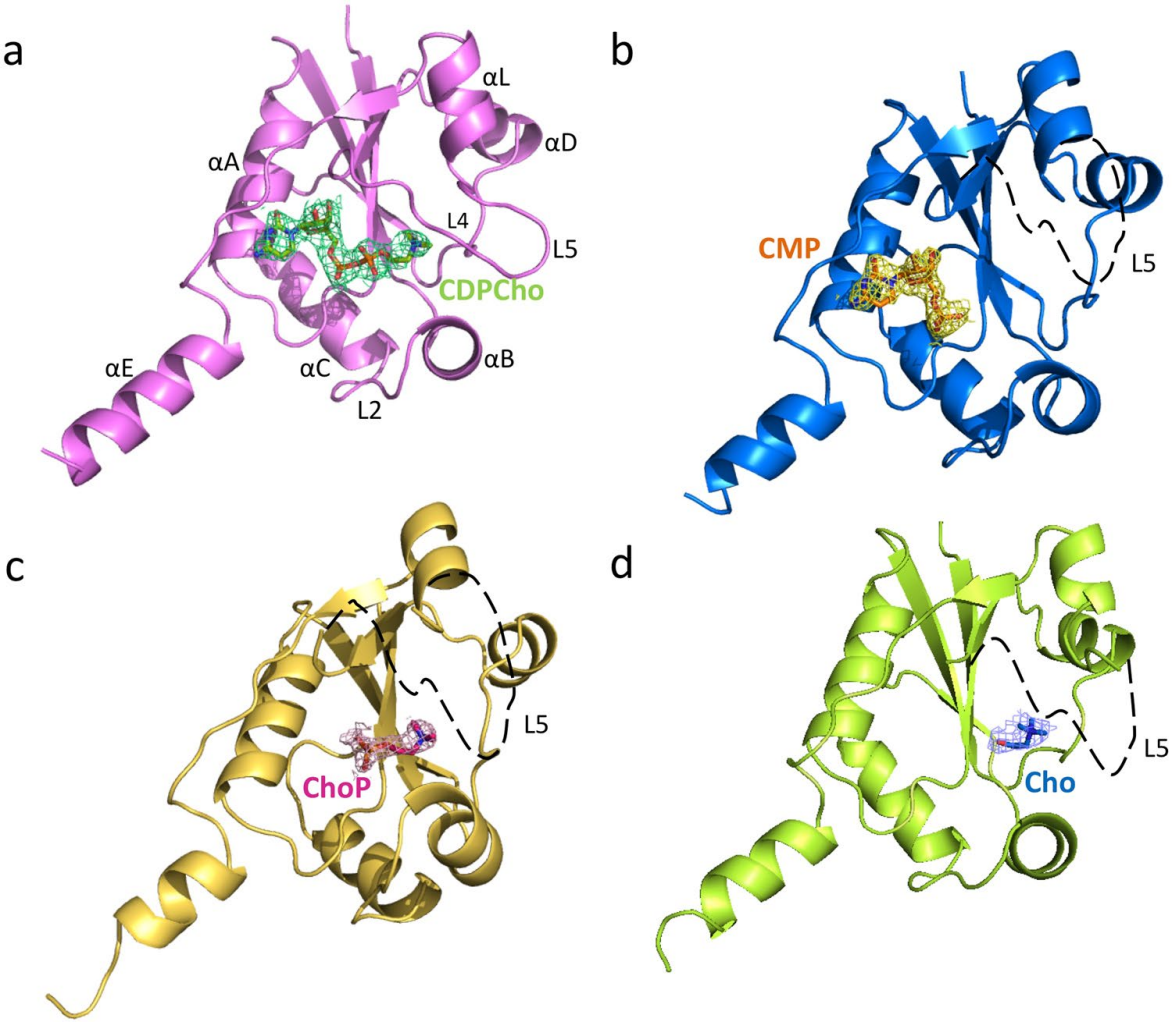
X-ray co-structures of CDPCho-*Pf*CCT, CMP-*Pf*CCT, ChoP-*Pf*CCT and Cho-*Pf*CCT monomers. Complexes with (**a**) CDPCho (PDB: 4ZCS) at 2.45 Å resolution, (**b**) CMP (PDB: 4ZCP) at 1.98 Å resolution, (**c**) ChoP (PDB: 4ZCR) at 1.80 Å resolution and (**d**) Cho (PDB: 4ZCQ) at 1.92 Å resolution. In all cases only one monomer of a dimer is presented. The ligands (CDPCho, CMP, ChoP and Cho) are shown in sticks with electron density around them. The 2F_o_ − F_c_ electron density omit maps were contoured at 1.0 sigma around the respective ligand within 1.6 Å of the selected atoms. The maps were created using *fft* function from CCP4 software^49^. The dashed black line in (**b**–**d**) represents loop L5 which is not visible for these co-structures.

**Figure 3:**
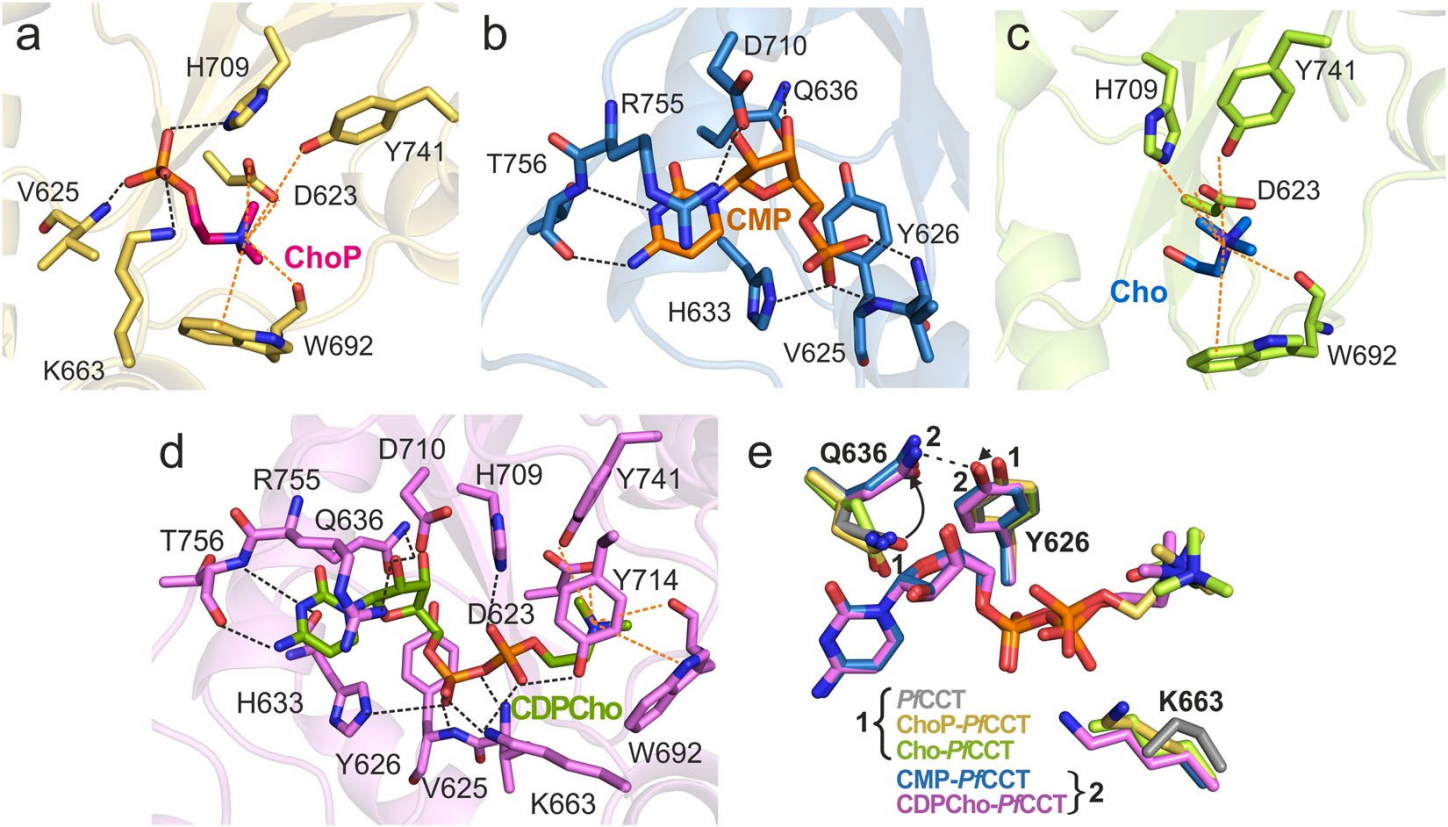
Ligand interactions and conformational changes of *Pf*CCT_(581–775)_ upon ligand binding. Close-up views of the substrate/product binding sites of *Pf*CCT. (**a**) CDPCho*-Pf*CCT structure, (**b**) CMP*-Pf*CCT structure, (**c**) ChoP*-Pf*CCT structure and (**d**) Cho*-Pf*CCT structure. Ligand interaction residues are labelled and showed in stick representation. Interactions with the nucleotide and choline moieties of ligands are represented by black and orange dashed lines, respectively. (**e**) Conformational changes of active site residues Y626, Q636 and K663 in different ligand complexes of *Pf*CCT_(581–775)_. Binding of nucleotide ligands induces a shift of Q636 and Y626 from state 1 to state 2. The K663 residue sidechain is not rendered fully visible in the free enzyme as well as in Cho and CMP complexes.

The 3D structures of *Pf*CCT_(581–775)_ in complex with CMP, Cho, ChoP or CDPCho (Table 3) possess almost identical overall folds as the free protein (Fig. 2 and Supplementary Table S1). In all structures, the ligands were found in the active site. The occupancy of ChoP was only 50% even when the crystal was obtained after soaking in very high (50 mM) final concentration of ChoP, which is in accordance to the very low affinity described above. Minor conformational changes were observed in loops L2 and L4 between strand β3 and helix αD. The major change concerned loop L5 (residues 712–738) that is only visible in the presence of CDPCho (Fig. 2a). In this case, ordered conformation of the loop is assisted by the interaction of Y714 with the ligand which is apparently not strong enough with ChoP or choline to render loop L5 ordered (Fig. 3). The nucleotide ligands CMP and CDPCho are anchored in the active site by several polar contacts, hydrogen bonds and a cation-π interaction of R755 with the cytosine ring (Fig. 3 and Supplementary Table S2). In contrast, the choline subsite that accommodates Cho, ChoP and the choline moiety of CDPCho provides ligand recognition with charged as well as cation-π interactions forming a composite aromatic box^30^. The coordination of the nucleophile phosphate moiety in ChoP- and CDPCho-liganded structures is ensured by V625, K663 and H709 residues. Analysis of these different liganded *Pf*CCT structures demonstrates conformational changes for a set of active site residues responsible for ligand accommodation, including D623, Y626, Q636, K663, W692, H709, Y714, I740, Y741 and R755.

### Deciphering the role of K663 in catalysis by structural, kinetic and thermodynamic analyses

K663 is a special active site residue that displays conformational variety in different liganded complexes (Fig. 3e). A catalytically essential role of its equivalent residue in *Rn*CCT (K122) had already been demonstrated using molecular dynamics (MD) simulations^24^ and mutagenesis studies^31^. Moreover, it was suggested that impeding its conformational dynamics has a major role in the enzyme regulatory mechanism^24^. Here, we found that in the free and the CMP-bound *Pf*CCT structures, the reliable positioning of the K663 sidechain is precluded due to the weak electron density, suggesting its high flexibility or disorder (Supplementary Fig. S3). The presence of Cho in the active site at least partially stabilizes the K663 side chain, while ChoP or CDPCho accommodation renders this residue fully visualized. The K663 terminal amino moiety points towards the phosphate group of ChoP or both phosphate moieties of CDPCho, in somewhat different conformations (Fig. 3e and Supplementary Fig. S3). These structural insights showing variable detection and interactions of K663 prompted us to an in-depth definition of its role throughout the course of the catalytic mechanism. We interrogated the functionality of this residue by site directed mutagenesis followed by biochemical analysis. Exchange of K663 to alanine rendered *Pf*CCT_(528–795)_ practically inactive (Table 1). The enzymatic activity was monitored by a coupled pyrophosphatase (PPase) assay and consisted of two consecutive steps: the cleavage of the byproduct pyrophosphate into two phosphates and the addition of phosphates to a nucleotide derivative, resulting a chromophore substance. Surprisingly, we observed a remnant enzymatic activity upon *Pf*CCT K663A enzyme addition, even in absence of both the ChoP substrate and the PPase auxiliary enzyme (Supplementary Fig. S4). The accumulation of free phosphate in the absence of the substrate, ChoP, argued for a CTP phosphohydrolase (CTP → CDP + P_i_) activity of the K663A mutant (Supplementary Fig. S4). Thus in a straightforward manner, we could conveniently distinguish the cytidylyltransferase from the CTP phosphohydrolase activities in our coupled enzyme assay for *Pf*CCT_(528–795)_ and its K663A mutant, respectively. For reliable characterization of ligand interactions of this lysine, we measured the equilibrium ligand binding of CDPCho and CTP to *Pf*CCT_(528–795)_ K663A (Table 2 and Supplementary Fig. S4). CDPCho binding affinity of the mutant suffered a four- to six-fold attenuation as evidenced by ITC measurements. An even more drastic effect was observed for CTP binding to K663A mutant where the reduced binding affinity was coupled with a fundamental change in the energetics of binding as compared to wild-type *Pf*CCT_(528–795)_. The observed endothermic character of binding (ΔH_CTP_ > 0) caused an entropy-favored binding mechanism that opposes the definitely exothermic interaction with the wild-type enzyme. This change can reflect either the increased conformational entropy due to a poorly defined binding conformation of the triphosphate of CTP or to a lesser extent, the solvent release upon binding to the mutated active site. Collectively, K663 appears to facilitate multiple steps in the catalytic mechanism. It has a decisive role in ChoP coordination as evidenced by its structural contacts (Supplementary Fig. S3 and Table S2) and by its absolute requirement for the classical CCT activity with ChoP as substrate. It also substantially contributes to the efficiency of CTP binding to the free enzyme.

### Comparison of *Pf*CCT and *Rn*CCT structures and functional characterization of residue alterations

**Figure 4:**
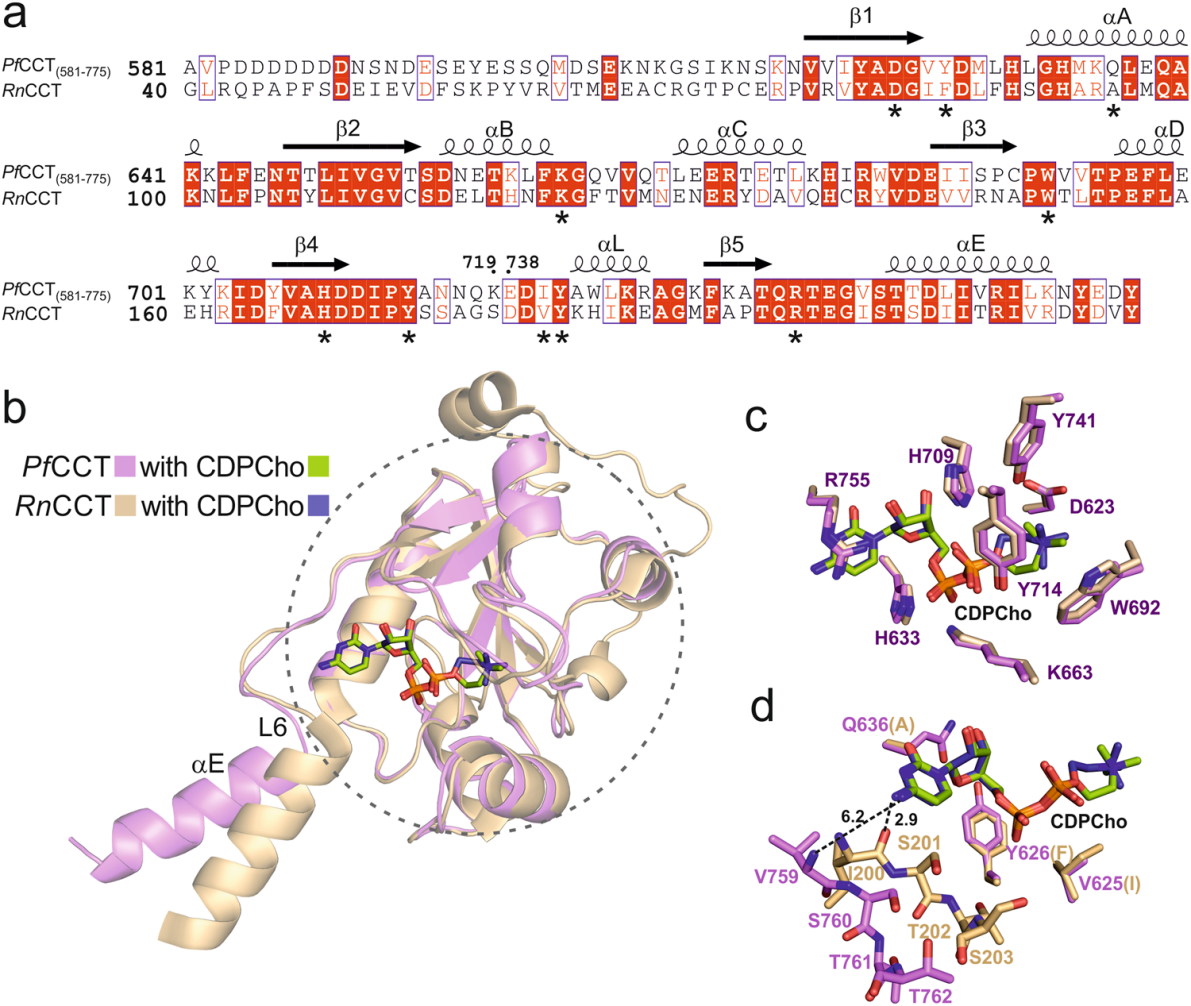
Structural comparison of *Pf*CCT and *Rn*CCT catalytic domain in complex with CDPCho. (**a**) Sequence alignment of *Pf*CCT_(581–775)_ and the corresponding region of *Rn*CCT. Strictly conserved residues are indicated as white letters on red background and similar residues as red letters on white background. The secondary structure elements are added above the sequence. We also noted by a star all residues mentioned in the text, including active site residues and non-conserved residues. Residue numbers are noted where the lysine-rich specific loop (720–737) has been removed. The layout was created by ESPript 3.0^57^. (**b**) Structural alignment of *Pf*CCT (violet) and *Rn*CCT (PDB: 3HL4) (light orange) monomers complexed with CDPCho. CDPCho is coloured by atoms with green and deep blue carbons in *Pf*CCT and *Rn*CCT complex structures, respectively. Dashed circle designates the core region which is highly similar in *Pf*CCT and *Rn*CCT structures. (**c**) Close-up view of the CDPCho interaction network. (**d**) Superposition of 3 non-conserved active site residues between *Pf*CCT and *Rn*CCT and displacement of the signature sequence fragment (*Pf*CCT: ^759^VSTT^762^; *Rn*CCT: ^200^ISTS^203^) located at the border of loop L6 and helix αE. Residues V625, Y626 and Q636 in *Pf*CCT correspond to I84, F85 and A95 in *Rn*CCT, respectively. Distances between atoms showed by dashed line are in Å.

The *Pf*CCT and *Rn*CCT catalytic domains share 44% sequence identity^23^ and high structural similarity (Fig. 4a,b). This especially holds for the core region of the conserved α/β Rossmann fold indicated by a dashed circle in Fig. 4b, that hosts the inner part of the active site accommodating CDPCho. This region, defined by residues 618–760 in *Pf*CCT displays an all-atom RMSD value of 0.73 Å with the respective region of the *Rn*CCT structure complexed with CDPCho^20^. CDPCho is found in the same zig-zag position in both *Pf*CCT and *Rn*CCT co-structures with only the two methylene groups of this ligand adopting different conformations. The interaction network of CDPCho is also largely similar (Fig. 4c). The differences in the catalytic site concern the residues V625, Y626, Q636 and V759 of *Pf*CCT corresponding to hydrophobic residues I84, F85, A95 and I200 of *Rn*CCT, respectively (Fig. 4d). A notable structural difference involves Q636 sidechain that enables a direct interaction with the ribose 3′OH moiety in *Pf*CCT, whereas a similar contact in *Rn*CCT is established by an ordered water molecule next to A95. Y626 and Q636 constitute the wall of the active site cavity around the hole for the ribose moiety of the ligands. Both residues display conformational changes during the course of catalysis (Fig. 3e). The position corresponding to Y626 is strictly reserved for aromatic (Y/F) residues in the HxGH nucleotidylyltransferase enzyme superfamily because the edge-on orientation of the aromatic sidechain secures the parallel conformation and hydrogen bond contacts of the two histidines from the HxGH motif, critical for their proper catalytic function^32^ (Supplementary Fig. S5). In a nucleotide-bound form, Q636 forms a hydrogen bridge to Y626, enabled by the phenolic OH group of the latter that is additionally present as compared to its *Rn*CCT surrogate F85. In order to evaluate the impact of these residue changes, we engineered a Y626F/Q636A double mutant in *Pf*CCT_(528–795)_ to mimic the *Rn*CCT enzyme. This construct displayed a six-fold attenuated catalytic rate together with an unaltered K_M,CTP_ value compared to *Pf*CCT_(528–795)_ (Table 1). Thermodynamic analysis revealed unaltered CTP affinity of the mutant with a slightly increased favorable binding enthalpy counterbalanced by a larger entropic penalty (Table 2). A modest effect of the residue substitutions was uncovered by the analysis of CDPCho binding. A somewhat tighter binding of CDPCho was observed in the case of the *Rn*CCT-mimicking construct *Pf*CCT_(528-795)_ Y626F/Q636A, accompanied by a decrease of both the favorable enthalpy and the unfavorable entropy components (Table 2). The differences observed for CDPCho binding to *Pf*CCT or to the *Rn*CCT-mimicking construct may be interpreted by a slightly more constrained CDPCho interaction in the wild-type *Pf*CCT enzyme. This is also supported by the reduced flexibility of the nucleotide binding pocket in the CDPCho-*Pf*CCT structure in comparison to the CDPCho-*Rn*CCT structure (PDB: 3HL4), as suggested by *B*-factor values.

### Conformational displacement at the PP_i_ binding region contributes to low activity of *Pf*CCT_(581–775)_

In contrast to the core region, the peripheral segments of the *Pf*CCT catalytic domain display considerable structural changes compared to *Rn*CCT. These include the N-terminal flexible segment of *Pf*CCT and the C-terminal protruding part consisting of loop L6 and helix αE (Fig. 4b). This C-terminal region contributes to the active site assembly by providing contacts between CTP and the strictly conserved CCT signature sequence RTEG(I/V)ST(S/T) (Supplementary Fig. S6). The terminal two residues of the signature sequence constitute the N-terminus of the helix αE and are expected to coordinate PP_i_ at the bottom of the active site^20^. The displacement of this functionally relevant part leads to the loss of contact between V759 main chain and the cytosine ring and to a ~5 Å displacement of T761 and T762 compared to what is observed in *Rn*CCT (Fig. 4d). To probe the functional role of these threonine residues, we designed two T/A single point mutants in *Pf*CCT_(528–795)_ and characterized changes in enzyme kinetics and CTP binding. We found that these alterations rendered the enzyme practically inactive, coupled with a substantial increase of K_M,CTP_ values from 170 µM for *Pf*CCT_(528–795)_ to 1,270 µM and 610 µM for *Pf*CCT_(528–795)_ T761A and *Pf*CCT_(528–795)_ T762A, respectively (Table 1). Nevertheless, analysis of CTP binding to these mutants followed by ITC reported a less substantial, three-fold attenuation effect on binding affinity (K_d,CTP_ values in Table 2). Collectively, kinetic analysis and ITC results suggest that while these threonine residues possibly contact CTP and PP_i_, they have a more prominent contribution to catalysis than to ligand binding.

## Discussion

**Figure 5:**
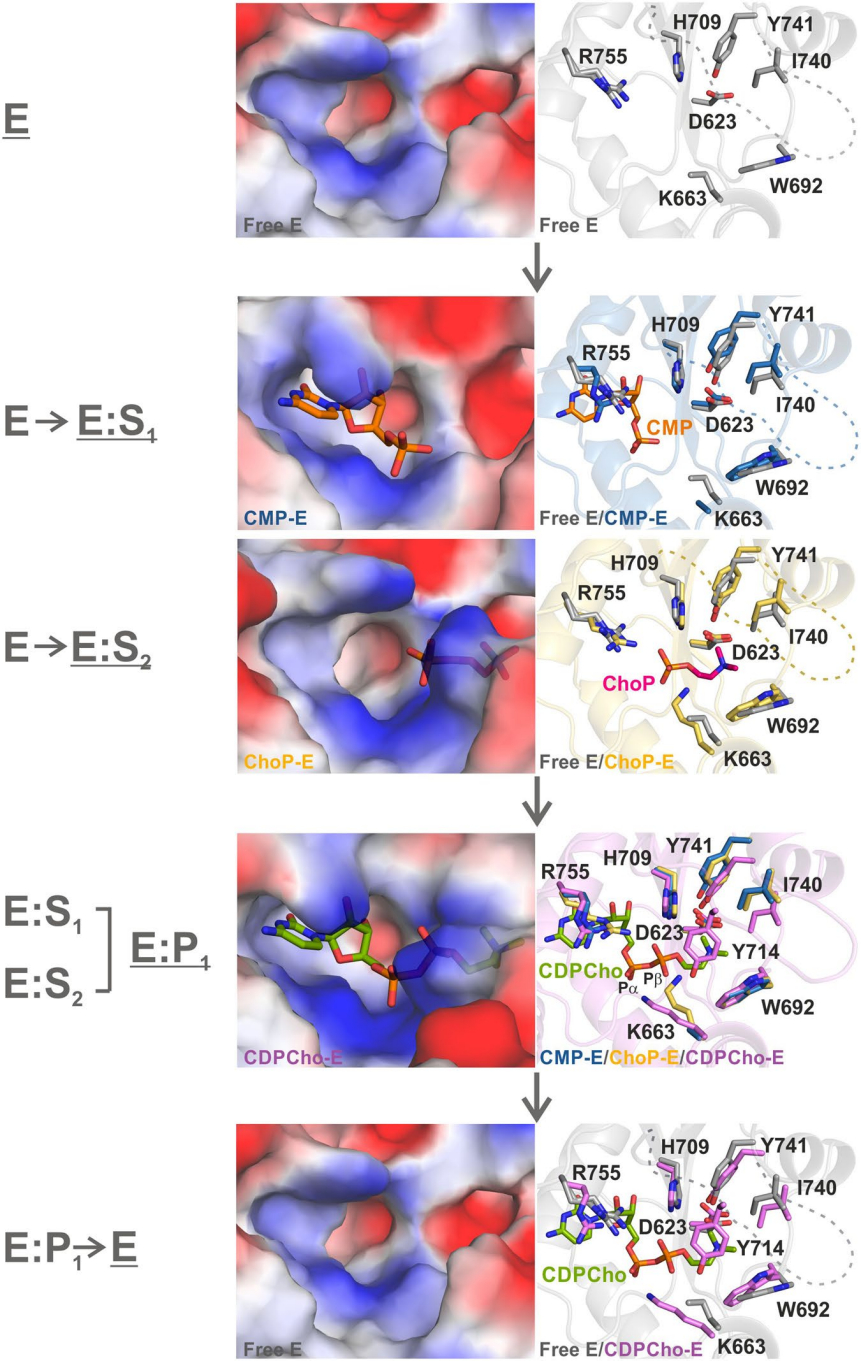
Structural views of *Pf*CCT active site at the different enzymatic steps. Electronic surface representations of the binding pockets (left) reveal polarity and cavity size changes along the enzymatic cycle. Close-up views of the active site (right) show key residues superimposed from two consecutive steps of the catalytic mechanism revealing the conformational changes upon substrate/product binding. Protein fold is shown in the background as cartoon for the later step. Residues from the free enzyme (E) are in grey, from the CMP-bound enzyme in blue, from the ChoP-bound enzyme in yellow and from the CDPCho-bound enzyme in violet. The ligands are colored as followed: CMP, orange (S_1_); ChoP, dark pink (S_2_) and CDPCho green (P_1_). In the free enzyme, R755 is found in two alternative conformations. Note that instead of the proper substrate CTP, CMP is present in the nucleotide subsite in E:S_1_ state. The α and β phosphate moieties of CDPCho are labelled on the relevant panel.

Prior to our study, only two high resolution structures of CCT catalytic domain and six structures of members of the Rossmann-folded cytidylyltransferase enzyme family were available from the Protein Data Bank. These include ECT (CTP:phosphoethanolamine cytidylyltransferase) and GCT (CTP:glycerol-3-phosphate cytidylyltransferase) catalytic domain structures that together with CCT share the conserved fold architecture as well as some key active site residues comprising the RTEG(I/V)ST(S/T) and HxGH signature sequences. All these enzymes are specialized in the cytidylyl transfer reaction with a hallmark of exploiting basic residues for substrate coordination and catalysis^31,33–35^. The herein presented structural data complemented with biochemical characterization provide an unprecedented step-by-step view of the enzymatic cycle of *Pf*CCT. The structural snapshots of subsequent steps of the catalytic mechanism readily identify steric fluctuations and conformational changes of individual residues at the choline and nucleotide subsites (Fig. 5). A limitation of the structural set originates from the C-terminal truncation of the catalytic domain with the removal of a part of the helix αE and the subsequent linker segment. It is very likely that this engineered modification is responsible for the altered orientation of the helix αE compared to CCT, ECT and GCT structures which leads to the detachment of its N-terminal T761 and T762 residues from the vicinity of the active site. We hypothesize that the apparent displacement of these catalytically important threonines may contribute to the low activity of the shortened *Pf*CCT_(581–775)_ construct used for crystallographic studies. We suspect that the helix αE in *Pf*CCT_(581–775)_ possesses significant conformational freedom in solution and that its observed conformation in the crystal structures is stabilized by crystal packing with local αE lattice contacts forming αE homotopic tetramers from different monomers (not shown).

The present structural set together with previously obtained data offers a stepwise understanding of the CCT catalytic mechanism (Fig. 5). Binding of CMP, a fragment of CTP substrate, to the free enzyme active site induces a major conformational change of R755 with a ~4 Å displacement to provide a cation-π stacking interaction with the cytosine group. A similar nucleotide ligand induced movement of the corresponding arginine has previously been observed by MD simulations for *Rn*CCT^24^ and is also seen in ECT and GCT co-structures. In case of *Pf*CCT, a flip of the non-conserved Q636 sidechain additionally provides space for the accommodation of the ribose. Notably, its alanine counterpart in *Rn*CCT lacks the ability for this sidechain flip. From a drug design perspective, targeting Q636 might enhance the selectivity to *Pf*CCT *versus* human CCT of a future chemical hit compound. The burying of the cytosine part is observed in all available cytidylyltransferase structures complexed with nucleotide ligands and is expected to contribute to the favorable binding enthalpy. ChoP binding to the choline subsite is accomplished by a slight inward movement of I740 and Y741 sidechains. Key residue contacts to ChoP that emerge from these movements involve the orientation of K663 towards the phosphate moiety and of W692 to provide cation-π interaction to the trimethylammonium moiety (Fig. 5). Loop L5 is not visible at this stage and is seen ordered only in the CDPCho product-bound form. The conformational fluctuations of this segment may constitute a side-entry of the active site that enables access of ChoP. While the disorder to order transition of loop L5 is first observed in the presented *Pf*CCT structures, the flexibility of this loop was also evidenced in *Rn*CCT by the comparatively high local *B-*factors as well as by fluctuations observed in MD simulations with or without CDPCho^24^. Intriguingly, loop L5 hardly establishes contacts to other structural elements, thus its anchoring to the active site is presumably fostered by the cation-π and polar interactions of Y714 with CDPCho (Fig. 5). Closure of the choline subsite in the CDPCho-bound state is also accompanied by the further inward movement of I740 and Y741. Meanwhile, K663 moves to coordinate the P_α_ group in addition to the P_β_ group of CDPCho that originated from ChoP, allowing the phenolic OH of Y714 to coordinate P_β_ moiety (Fig. 5). K663 may also be involved in the formation of a productive ES_1_S_2_ ternary complex and in the catalytic step. Its position and function during the transition state remains to be resolved. It may either coordinate nucleophile phosphate of ChoP or provide a bridge between the two phosphate moieties directly involved in catalysis, a conformation apparent in CDPCho liganded *Pf*CCT and *Rn*CCT structures and reminiscent of the position of K46 in GCT^34^. So far no CCT structure complexed to CTP is available. Structural insights of related GCT:CTP^36^ and ECT:CTP (PDB: 4XSV) complexes reveal that the serine and threonine residues corresponding to T761 and T762 of *Pf*CCT participate in the coordination of CTP phosphate moieties. The mutation into alanine of the threonine residue of *Bs*GCT, equivalent to *Pf*CCT T761, results in a pronounced decrease of the enzyme activity^37^. Our biochemical results indicate the importance of these threonine residues in CTP binding and catalysis. Interestingly, MD simulations show that the binding of the AI helix represses the dynamics of the Lys122 of *Rn*CCT (Lys663 of *Pf*CCT) but also of helix αE which includes serine/threonine counterparts of T761 and T762^24.27^.

In conclusion, we provide novel structural insights into the *Pf*CCT-catalyzed enzymatic steps that are supported by biochemical characterization of key elements in CCT action. A detailed knowledge at the molecular level of the catalytic mechanism of *Pf*CCT constitutes the first step to the rational identification of efficient and specific inhibitors. The PC biosynthesis pathway is already validated as an antimalarial target at the parasite blood-stage^15,38^. Recently, the activity of this pathway has also been directly linked to *P. falciparum* transmission-stage formation^11^. Inhibiting *Pf*CCT, the key enzyme of PC pathway, should therefore have a strong impact at multiple stages of the parasite development.

## Methods

### Cloning, bacterial expression and purification of CCT constructs

The *Pf*CCT cDNA sequence (PlasmoDB: PF3D7_1316600) was codon-optimized for expression in *E. coli* (GenScript). The *Pf*CCT_(581–775)_ construct was obtained using previously described *Pf*CCT_(528–795)_ lacking the lysine-rich *Plasmodium* specific loop (720–737)^16^. The DNA fragment was cloned into the expression vector pET15bTEV (modified from pET15b, Novagen) using the NdeI/BamHI sites in order to produce N-terminal 6 × His-tagged protein where the 6 × His-tag is cleavable by tobacco etch virus (TEV) protease. The Y626F/Q636A double mutant, the T761A, T762A and the K663A mutant constructs of His-tagged *Pf*CCT_(528–795)_ were produced by site-directed mutagenesis using the QuikChange method (Agilent). Primers were synthesized by Eurofins MWG GmbH. Primer sequences are given in Supplementary Table S3. *E. coli* BL21 (DE3) cells transformed with the plasmid were grown in Luria-Bertani (LB) broth containing 100 mg.ml^−1^ ampicillin at 37 °C to OD = 0.6. Protein expression was induced by 0.5 mM IPTG and bacteria were continuously cultured for 24 h at 16 °C. Cells were harvested and re-suspended in lysis buffer containing 20 mM Tris/HCl pH 7.5, 0.15 M NaCl and 2 mM ethanethiol (EtSH). Supernatant containing protein was obtained by lysing cells using a French press, followed by centrifugation at 40 000 *g* for 1 h. The supernatant was loaded onto a HisTrap HP column (GE Healthcare) pre-equilibrated with lysis buffer. His-*Pf*CCT_(581–775)_ was eluted with 150 mM and 250 mM imidazole in lysis buffer. Removal of the His_6_-tag was performed after the affinity column by incubation with recombinant His_6_-TEV protease for one night at room temperature (protease:protein ratio of 1:100 w-w). After concentration, 1 ml of protein sample was further purified by gel filtration using a HiLoad 16/60 Superdex 200 pg (GE Healthcare) in lysis buffer. Purification of His-tagged *Pf*CCT_(528–795)_ mutant constructs is described in Nagy *et al*.^16^.

### Enzyme kinetics

Enzyme activity measurements were performed as described previously, using a continuous coupled colorimetric assay with pyrophosphatase (PPase), purine nucleoside phosphorylase (PNP) auxiliary enzymes and the auxiliary substrate 7-methyl-6-thioguanosine (MESG)^16,30,39^. For CTP substrate titrations, CTP concentration was varied between 100 μM and 2 mM while ChoP concentration was kept constant at 5 mM. For ChoP titrations, ChoP concentration was varied between 0.5 mM and 20 mM while CTP concentration was kept constant at 1 mM. Initial velocities were calculated from the first 10% of the progress curves using phosphate calibration as reference^16^. Due to the low activity of *Pf*CCT_(581–775)_ and *Pf*CCT_(528–795)_ T761A, T762A variants, the enzyme was used at 10 μM concentration and the slope of the absorbance change was determined after monitoring the reaction for 20 minutes. For measurements with *Pf*CCT_(528–795)_ K663A the following reagent concentrations were used in the enzyme assay: [PPase] = 0.17 U·ml^−1^, [PNP] = 1.25 U·ml^−1^, [MESG] = 0.1 mM, [Mg^2+^] = 5 mM and [*Pf*CCT_(528–795)_ K663A] = 10 μM, to yield adequate signal-to-noise ratio (S/N > 10). Three independent experiments were performed for CTP and ChoP kinetic titrations of *Pf*CCT_(528–795)_ K663A. Global fit of CTP and ChoP kinetic titrations for each enzyme variant was performed in Origin 8.6 using Michaelis–Menten equation, to yield a single k_cat_ parameter.

### ITC measurements

Calorimetric measurements were performed on an ITC 200 titration calorimeter (Malvern) at 20 °C. The protein samples were dialyzed against 20 mM HEPES/NaOH pH 7.5, 100 mM NaCl, including 0.5 mM Tris(2-carboxyethyl)phosphine (TCEP) as reducing agent. CTP and CDPCho were diluted from a concentrated stock into the dialysis buffer with subsequent pH adjustment. Concentration of the titrating nucleotide ligands were determined using molar extinction coefficients 9000 M^−1^ cm^−1^ at 271 nm for CTP and 5000 M^−1^ cm^−1^ at 260 nm for CDPCho^16^. In the experimental setup, the cell of the instrument was filled with protein and the syringe with the ligand. The titrations typically included 19 steps of injection with 2 μl of ligand per injection spaced 180 s apart from each other, with the injection syringe rotating at 750 r.p.m. The data were analyzed using MICROCAL ORIGIN software following the directions of the manufacturer. To account for dilution, the invariant integrated heat value of the last 3–5 data points was subtracted from all data points. The one set of independent sites binding model was applied to data for determination of thermodynamic parameters: dissociation constant (K_d_), stoichiometry (N), enthalpy (ΔH) and entropy (ΔS). Due to the low apparent affinity, the N value was fixed to a constant value of 1 in CTP titrations to *Pf*CCT_(528–795)_ K663A according to the low c value ITC data analysis protocol^40,41^. For CTP and CDPCho titrations of *Pf*CCT_(528–795)_ K663A, and CTP titrations of T761A and T762A variants of *Pf*CCT_(528-795)_, mean and SD from two independent experiments are provided, whereas for CDPCho titrations of *Pf*CCT_(581–775)_ and *Pf*CCT_(528-795)_ mean and SD from three independent experiments are provided. For *Pf*CCT_(528-795)_ (Y626F/Q636A), errors of model fit to data of a single ligand binding experiment are given.

### 2D ^1^H NMR Spectroscopy

NMR experiments for the assignments of the flexible part of *Pf*CCT_(581–775)_ were recorded at 283 K on a Bruker 700 MHz NMR spectrometer equipped with a cryogenic 5 mm Z-gradient probe head. The aqueous samples were containing 0.5 mM unlabeled *Pf*CCT_(581–775)_ in 20 mM Tris/HCl pH 7.5, 150 mM NaCl and 2 mM EtSH in 10% D_2_O as an internal lock. Two-dimensional ^1^H-^1^H TOCSY (isotropic mixing: 60 ms) and ^1^H-^1^H NOESY (mixing time: 200 ms) were performed for the assignments of the backbone and aliphatic side chain ^1^H resonances. Water suppression was achieved with the WATERGATE sequence^42^ in both experiments. NMR spectra were processed using GIFA software^43^.

### Crystallization and diffraction data collection

10 mg/ml of *Pf*CCT_(581-775)_ with 20 mM of CDPCho (final protein concentration 8 mg/ml) in the lysis buffer were screened by commercially available Classics screen (NeXtal, Qiagen) by sitting drop method. 200 nl sitting drops were set up using a 1:1 ratio between protein and precipitant in 96-well Greiner crystallization plates using a Cartesian dispensing robot^44^. After one month crystals of protein-CDPCho complex appeared in 0.2 M NaF, 0.1 M HEPES pH 7.5, 20% PEG 3350 at 20 °C. The ligand-free *Pf*CCT crystals were grown by the hanging-drop vapor-diffusion method at 4 °C by mixing 1 µl of protein solution at concentration of 10 mg/ml and 1 µl reservoir solution (0.08 M NaF, 0.1 M Tris pH 7.5, 24% PEG 3350) and appeared after 3 weeks. CMP-bound and ChoP-bound protein crystals were obtained by soaking crystals with CMP and ChoP dissolved in lysis buffer (20 mM Tris/HCl pH 7.5, 0.15 M NaCl and 2 mM EtSH). To obtain these co-crystals, ligand-free crystals were first grown by the hanging-drop vapor-diffusion method at 20 °C by mixing 1 µl of protein solution at concentration 8 mg/ml and 1 µl reservoir solution (pH 8.0, 20% PEG 4000). Crystals appeared after 2 months and were soaked with 100 mM CMP and 100 mM ChoP (50 mM final concentration) for 5 days and 4 weeks, respectively. Cho-bound crystals were obtained by the hanging-drop vapor-diffusion method in 0.08 M NaF, 0.1 M Tris pH 7.5, 24% PEG 3350 at 20 °C by co-crystallization with 50 mM Cho dissolved in the lysis buffer. Crystals appeared after one week and were left to grow for an additional three weeks. Crystals were mounted and soaked in oil as cryosolvent, then frozen in liquid nitrogen. Diffraction data were collected at beamlines ID29, ID23-1 and ID23-2 of the European Synchrotron Radiation Facilities in Grenoble, France. These crystals belong to space group I_222_ with the asymmetric unit containing one monomer in the case of the free, CMP-bound, ChoP-bound and Cho-bound forms, and six monomers in the case of the CDPCho-bound form.

### Structure determination and refinement

The indexing and integration of obtained data sets were carried out using XDS (Pilatus)^45,46^. The scaling, merging and calculating of structural factor amplitudes were executed using programs TRUNCATE^47^ and SCALA^48^ from CCP4 software^49^. Crystal structures were constructed by molecular replacement using Phaser^50^ with *Rn*CCT (PDB: 3HL4) and free *Pf*CCT (PDB: 4ZCT) as search models, followed by model building in Coot^51^. The structure refinements were done using REFMAC 5.8.0073^52,53^ and PHENIX 1.9.1692^54^. Molecular graphics representations were created using PyMol^55^. All the ligand structures were created using PRODRG server^56^. Crystal diffraction data and final refinement statistics were summarized in Table 3.

### Accession codes

Atomic coordinates and structure factors for the reported crystal structures have been deposited in the PDB under ID codes 4ZCT, 4ZCP, 4ZCQ, 4ZCR and 4ZCS for the free, CMP-, Cho-, ChoP- and CDPCho-bound *Pf*CCT, respectively.

## Electronic supplementary material

Supplementary Information

## References

[1] White NJ (2014). Malaria. Lancet.

[2] WHO. World Malaria Report 2016. *World Health Organization*, 1–186 (2016).

[3] Breman JG (2012). Resistance to artemisinin-based combination therapy. Lancet Infect. Dis..

[4] Dondorp AM (2009). Artemisinin resistance in Plasmodium falciparum malaria. N. Engl. J. Med..

[5] Botte CY (2013). Atypical lipid composition in the purified relict plastid (apicoplast) of malaria parasites. Proc. Natl. Acad. Sci. USA.

[6] Dechamps S, Shastri S, Wengelnik K, Vial HJ (2010). Glycerophospholipid acquisition in Plasmodium - a puzzling assembly of biosynthetic pathways. Int. J. Parasitol..

[7] Vial, H. *et al*. In *Apicomplexan Parasites: Molecular Approaches toward* Targeted Drug *Development* (ed Katja Becker) 137–162 (Wiley-VCH Press, 2011).

[8] Vial HJ (2004). Prodrugs of bisthiazolium salts are orally potent antimalarials. Proc. Natl. Acad. Sci. USA.

[9] Wein S (2017). High Accumulation and *In Vivo* Recycling of the New Antimalarial Albitiazolium Lead to Rapid Parasite Death. Antimicrob Agents Chemother.

[10] Wengelnik K (2002). A class of potent antimalarials and their specific accumulation in infected erythrocytes. Science.

[11] Brancucci NMB (2017). Lysophosphatidylcholine Regulates Sexual Stage Differentiation in the Human Malaria Parasite Plasmodium falciparum. Cell.

[12] Dechamps S (2010). The Kennedy phospholipid biosynthesis pathways are refractory to genetic disruption in Plasmodium berghei and therefore appear essential in blood stages. Mol. Biochem. Parasitol..

[13] Sen P, Vial HJ, Radulescu O (2013). Kinetic modelling of phospholipid synthesis in Plasmodium knowlesi unravels crucial steps and relative importance of multiple pathways. BMC systems biology.

[14] Gonzalez-Bulnes P (2011). PG12, a phospholipid analog with potent antimalarial activity, inhibits Plasmodium falciparum CTP:phosphocholine cytidylyltransferase activity. J. Biol. Chem..

[15] Wein S (2012). Transport and pharmacodynamics of albitiazolium, an antimalarial drug candidate. Br. J. Pharmacol..

[16] Nagy GN (2013). Evolutionary and mechanistic insights into substrate and product accommodation of CTP:phosphocholine cytidylyltransferase from Plasmodium falciparum. FEBS J..

[17] Park YS, Sweitzer TD, Dixon JE, Kent C (1993). Expression, purification, and characterization of CTP:glycerol-3-phosphate cytidylyltransferase from Bacillus subtilis. J. Biol. Chem..

[18] Sundler R (1975). Ethanolaminephosphate cytidylyltransferase. Purification and characterization of the enzyme from rat liver. J. Biol. Chem..

[19] Veitch DP, Gilham D, Cornell RB (1998). The role of histidine residues in the HXGH site of CTP:phosphocholine cytidylyltransferase in CTP binding and catalysis. Eur. J. Biochem..

[20] Lee J, Johnson J, Ding Z, Paetzel M, Cornell RB (2009). Crystal structure of a mammalian CTP: phosphocholine cytidylyltransferase catalytic domain reveals novel active site residues within a highly conserved nucleotidyltransferase fold. J. Biol. Chem..

[21] Cornell RB (2016). Membrane lipid compositional sensing by the inducible amphipathic helix of CCT. Biochim. Biophys. Acta.

[22] Cornell RB, Ridgway ND (2015). CTP:phosphocholine cytidylyltransferase: Function, regulation, and structure of an amphitropic enzyme required for membrane biogenesis. Prog. Lipid Res..

[23] Contet A (2015). Plasmodium falciparum CTP:phosphocholine cytidylyltransferase possesses two functional catalytic domains and is inhibited by a CDP-choline analog selected from a virtual screening. FEBS Lett..

[24] Lee J, Taneva SG, Holland BW, Tieleman DP, Cornell RB (2014). Structural basis for autoinhibition of CTP:phosphocholine cytidylyltransferase (CCT), the regulatory enzyme in phosphatidylcholine synthesis, by its membrane-binding amphipathic helix. J. Biol. Chem..

[25] Ding Z (2012). A 22-mer segment in the structurally pliable regulatory domain of metazoan CTP: phosphocholine cytidylyltransferase facilitates both silencing and activating functions. J. Biol. Chem..

[26] Huang HK (2013). The membrane-binding domain of an amphitropic enzyme suppresses catalysis by contact with an amphipathic helix flanking its active site. J. Mol. Biol..

[27] Ramezanpour M, Lee J, Taneva SG, Tieleman DP, Cornell RB (2018). An auto-inhibitory helix in CTP:phosphocholine cytidylyltransferase hijacks the catalytic residue and constrains a pliable, domain-bridging helix pair. J. Biol. Chem..

[28] Cornell RB (1995). Functions of the C-terminal domain of CTP: phosphocholine cytidylyltransferase. Effects of C-terminal deletions on enzyme activity, intracellular localization and phosphorylation potential. Biochem. J..

[29] Yang W, Boggs KP, Jackowski S (1995). The association of lipid activators with the amphipathic helical domain of CTP:phosphocholine cytidylyltransferase accelerates catalysis by increasing the affinity of the enzyme for CTP. J. Biol. Chem..

[30] Nagy GN (2014). Composite aromatic boxes for enzymatic transformations of quaternary ammonium substrates. Angew. Chem. Int. Ed. Engl..

[31] Helmink BA, Braker JD, Kent C, Friesen JA (2003). Identification of lysine 122 and arginine 196 as important functional residues of rat CTP:phosphocholine cytidylyltransferase alpha. Biochemistry.

[32] D’Angelo I (2000). Structure of nicotinamide mononucleotide adenylyltransferase: a key enzyme in NAD(+) biosynthesis. Structure.

[33] Maheshwari S (2013). Biochemical characterization of Plasmodium falciparum CTP:phosphoethanolamine cytidylyltransferase shows that only one of the two cytidylyltransferase domains is active. Biochem. J..

[34] Pattridge KA (2003). Glycerol-3-phosphate cytidylyltransferase. Structural changes induced by binding of CDP-glycerol and the role of lysine residues in catalysis. J. Biol. Chem..

[35] Tian S (2014). Human CTP:phosphoethanolamine cytidylyltransferase: enzymatic properties and unequal catalytic roles of CTP-binding motifs in two cytidylyltransferase domains. Biochem. Biophys. Res. Commun..

[36] Weber CH, Park YS, Sanker S, Kent C, Ludwig ML (1999). A prototypical cytidylyltransferase: CTP:glycerol-3-phosphate cytidylyltransferase from bacillus subtilis. Structure.

[37] Park YS (1997). Identification of functional conserved residues of CTP:glycerol-3-phosphate cytidylyltransferase. Role of histidines in the conserved HXGH in catalysis. J. Biol. Chem..

[38] Peyrottes S (2012). Choline analogues in malaria chemotherapy. Curr. Pharm. Des..

[39] Marton L (2015). Molecular Mechanism for the Thermo-Sensitive Phenotype of CHO-MT58 Cell Line Harbouring a Mutant CTP:Phosphocholine Cytidylyltransferase. PLoS One.

[40] Tellinghuisen J (2008). Isothermal titration calorimetry at very low c. Anal. Biochem..

[41] Turnbull WB, Daranas AH (2003). On the value of c: can low affinity systems be studied by isothermal titration calorimetry?. J. Am. Chem. Soc..

[42] Piotto M, Saudek V, Sklenar V (1992). Gradient-tailored excitation for single-quantum NMR spectroscopy of aqueous solutions. J. Biomol. NMR.

[43] Pons JL, Malliavin TE, Delsuc MA (1996). Gifa V. 4: A complete package for NMR data set processing. J. Biomol. NMR.

[44] Walter TS (2005). A procedure for setting up high-throughput nanolitre crystallization experiments. Crystallization workflow for initial screening, automated storage, imaging and optimization. Acta Crystallogr. D Biol. Crystallogr..

[45] Kabsch W (1993). Automatic processing of rotation diffraction data from crystals of initially unknown symmetry and cell constants. J. Appl. Crystallogr..

[46] Kabsch W (2010). Xds. Acta Crystallogr. D Biol. Crystallogr..

[47] French S, Wilson K (1978). On the treatment of negative intensity observations. Acta Crystallogr. Sect. A.

[48] Evans P (2006). Scaling and assessment of data quality. Acta Crystallogr. D Biol. Crystallogr..

[49] Winn MD (2011). Overview of the CCP4 suite and current developments. Acta Crystallogr. D Biol. Crystallogr..

[50] McCoy AJ (2007). Phaser crystallographic software. J. Appl. Crystallogr..

[51] Emsley P, Cowtan K (2004). Coot: model-building tools for molecular graphics. Acta Crystallogr. D Biol. Crystallogr..

[52] Murshudov GN, Vagin AA, Dodson EJ (1997). Refinement of macromolecular structures by the maximum-likelihood method. Acta Crystallogr. D Biol. Crystallogr..

[53] Murshudov GN (2011). REFMAC5 for the refinement of macromolecular crystal structures. Acta Crystallogr. D Biol. Crystallogr..

[54] Adams PD (2010). PHENIX: a comprehensive Python-based system for macromolecular structure solution. Acta Crystallogr. D Biol. Crystallogr..

[55] DeLano, W. L. The PyMOL Molecular Graphics System. http://www.pymol.org, DeLano Scientific, San Carlos, CA, USA (2002).

[56] Schüttelkopf AW, van Aalten DMF (2004). PRODRG: a tool for high-throughput crystallography of protein-ligand complexes. Acta Crystallogr. Section D.

[57] Robert X, Gouet P (2014). Deciphering key features in protein structures with the new ENDscript server. Nucleic Acids Res..

