# Supplementary material for: Structural determinants of the catalytic mechanism of Plasmodium CCT, a key enzyme of malaria lipid biosynthesis
Source: Sci Rep. 2018 Jul 25;8:11215. doi: 10.1038/s41598-018-29500-9 (PMC6060094; doi:10.1038/s41598-018-29500-9)
Supplement: Supplementary file 1 — Supplementary Information [file 41598_2018_29500_MOESM1_ESM.pdf]

SUPPLEMENTARY INFORMATION

Structural determinants of the catalytic mechanism of *Plasmodium* CCT, a key enzyme of malaria lipid biosynthesis

Ewelina Guca[1,†], Gergely N. Nagy[2,3,‡], Fanni Hajdú[2,3], Lívia Marton[3,4], Richard Izrael[2,3], François Hoh[5,6], Yinshan Yang[5,6], Henri Vial[1], Beata G. Vertessy[2,3], Jean-François Guichou[5,6] and Rachel Cerdan[1*]

[1]Dynamique des Interactions Membranaires Normales et Pathologiques, UMR 5235, CNRS, Université de Montpellier, Montpellier, France

[2] Department of Applied Biotechnology and Food Science, Budapest University of Technology and Economics, Budapest, Hungary

[3] Institute of Enzymology, Research Centre for Natural Sciences, Hungarian Academy of Sciences, Budapest, Hungary

[4] Doctoral School of Multidisciplinary Medical Science, University of Szeged, Szeged, Hungary

[5]CNRS UMR5048, Centre de Biochimie Structurale, Université de Montpellier, Montpellier, France

[6] INSERM U1054, Montpellier, France

[†]Present address: Institute for Research in Biomedicine, The Barcelona Institute of Science and Technology, Carrer de Baldiri Reixac 10, 08028 Barcelona, Spain.

[‡]Present address: Division of Structural Biology, University of Oxford, Roosevelt Drive, Oxford OX37BN, United Kingdom

[*]corresponding. rachel.cerdan@umontpellier.fr

Figure S1

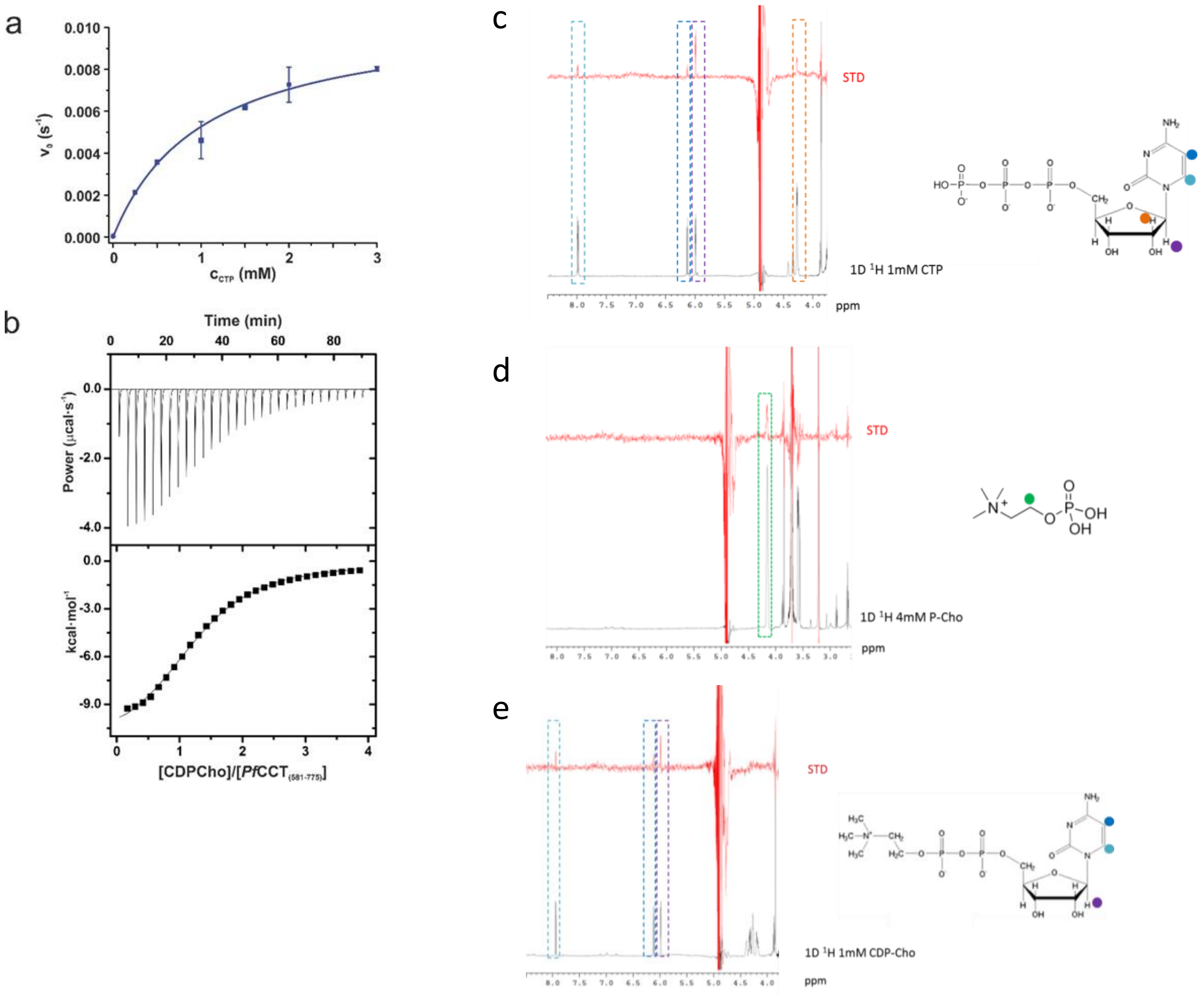

Supplementary Figure S1. Enzyme activity and ligand binding to *Pf*CCT$_{(581-775)}$ followed by ITC and STD (**a**) CTP kinetic titration of *Pf*CCT$_{(581-775)}$ at a fixed ChoP concentration of 5 mM. Kinetic data are fitted with the Michaelis–Menten equation. (**b**) Equilibrium CDPCho binding to *Pf*CCT$_{(581-775)}$ followed by ITC. Titration of 297 µM *Pf*CCT$_{(581-775)}$ with 6 mM CDPCho performed at 20°C is shown. Values of the measured kinetic and thermodynamic parameters and their errors from regression analysis are given in Table 1. (**c**), (**d**) and (**e**) STD spectra (red) and 1D ^{1}H spectra (black) of 50 µM *Pf*CCT$_{(581-775)}$ in 20 mM Tris/HCl pH 7.5, 150 mM NaCl and 2 mM EtSH are shown in presence of **c**) 1 mM CTP, **d**) 4 mM ChoP and **e**) 1 mM CDPCho. On the right, the protons highlighted by colored dots on chemical structures correspond to the resonances showing STD signal in colored frames on the left. The 1D ^{1}H STD-NMR spectra were recorded on a Bruker 500 MHz instrument at 283 K with 256 scans and selective saturation of protein resonances at 0.922 ppm using a series of Gaussian shaped pulses (20 ms, 1 ms delay between the pulses). The experiments were conducted with addition of 10% D_2O. All 1D ^{1}H NMR spectra were recorded with 16 scans.

Figure S2

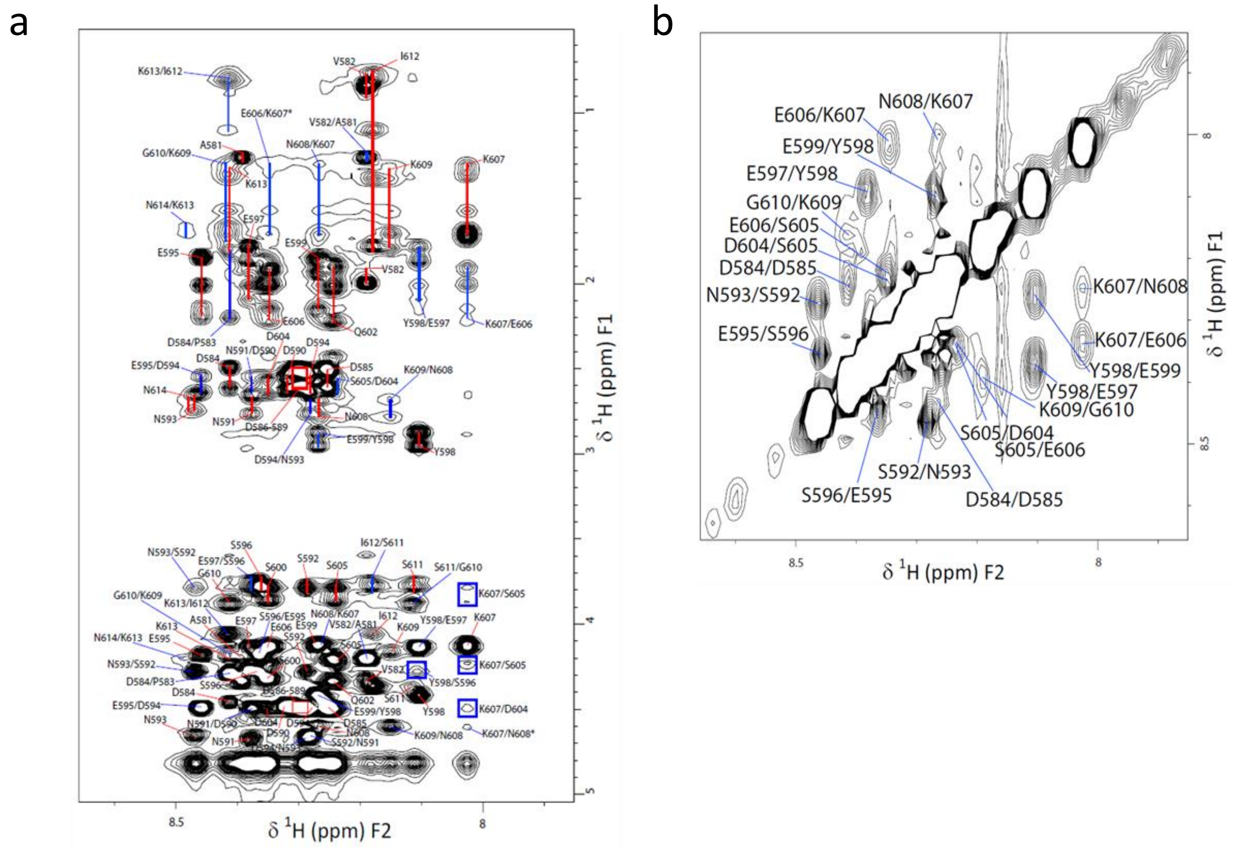

Supplementary Figure S2. NOESY 2D ^{1}H-^{1}H spectrum of 0.5 mM *Pf*CCT$_{(581\text{-}775)}$ indicates the presence of a 33-residue-long N-terminal disordered segment. (**a**) NH – H aliphatic region with intra-residue cross-peaks and inter-residue cross-peaks highlighted with red and blue colors, respectively. Blue squares show cross-peaks indicating the presence of the secondary structure. (**b**) NH – NH region. Cross-peaks suggest the presence of an α-helical segment from D604 to G610 and a short α-turn (E597, Y598 and E599).

Figure S3

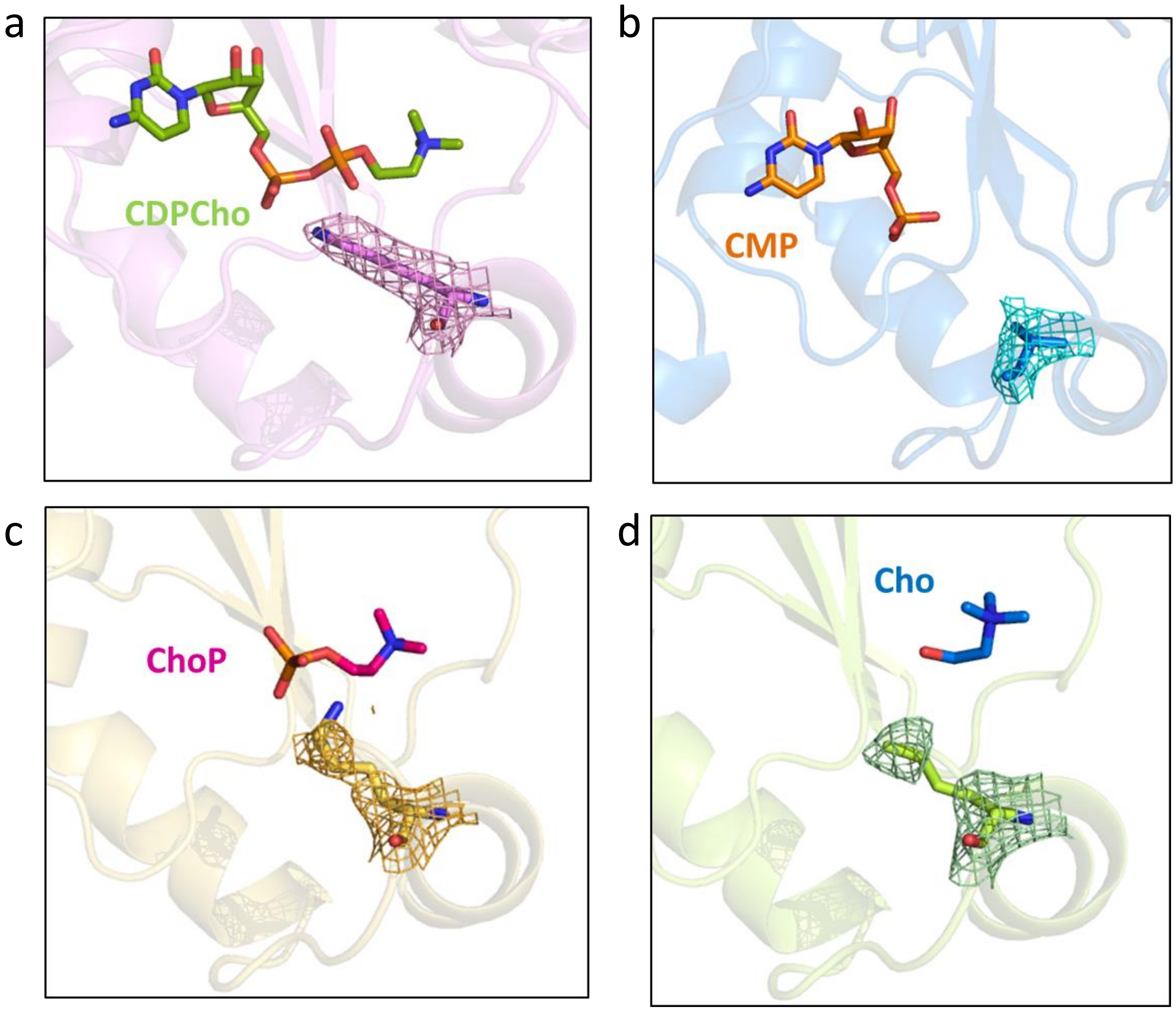

Supplementary Figure S3. Orientation of K663 in different ligand bound *Pf*CCT structures. Ligands are depicted as sticks in the active site pocket: (**a**) CDPCho, (**b**) CMP, (**c**) ChoP, (**d**) Cho. K663 is shown in sticks with electron density around the residue. The $2F_o$-F_c electron density maps were contoured at 1.0 sigma around K663 within 1.4 Å of the selected atoms. The maps were created using *fft* function from CCP4 software[1].

Figure S4

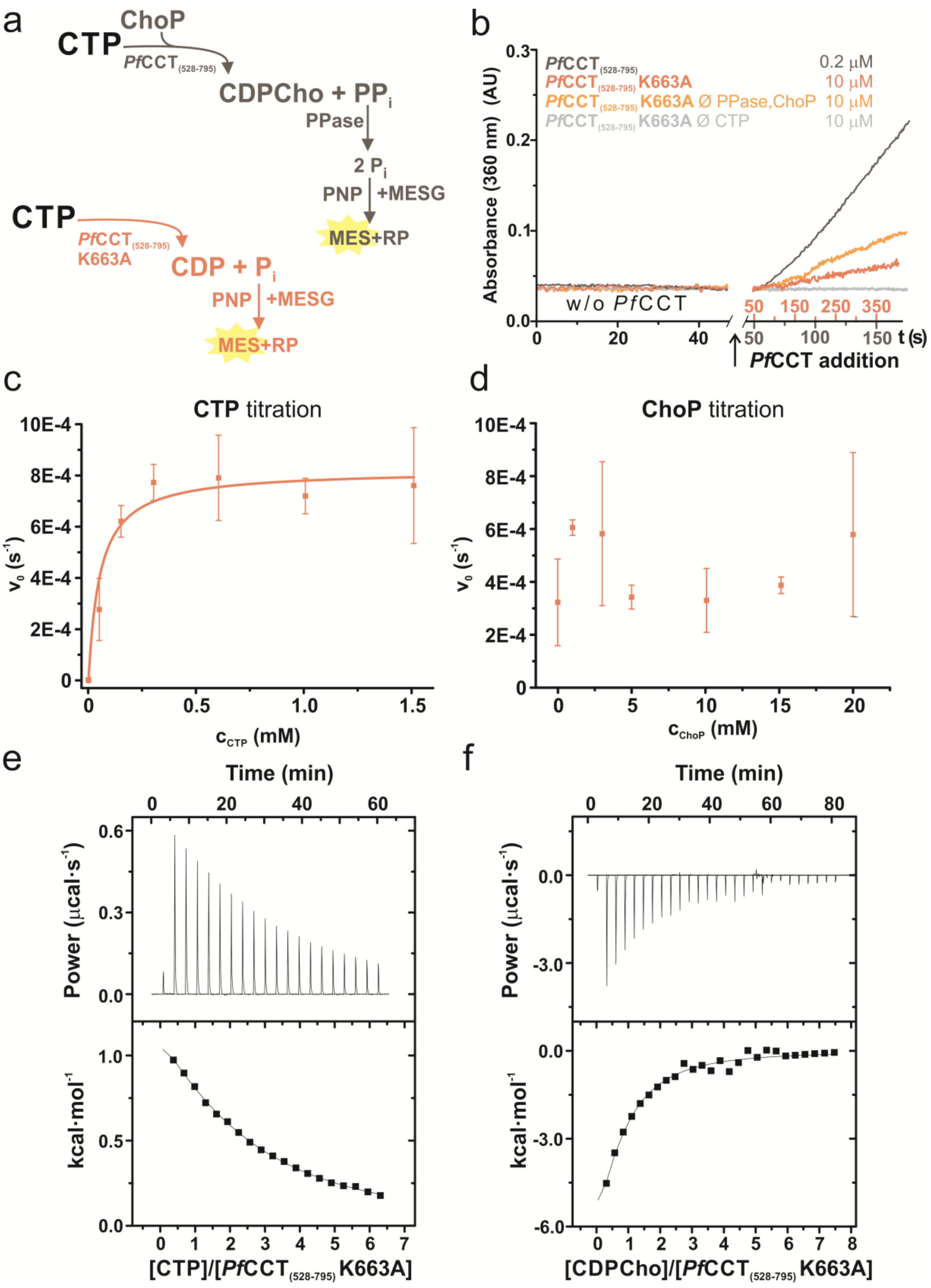
a
CTP
ChoP
PfCCT(528-795)
CDPCho + PPi
PPase
2 Pi
PNP +MESG
MES+RP
CTP
PfCCT(528-795) K663A
CDP + Pi
PNP +MESG
MES+RP
b
PfCCT(528-795) 0.2 µM
PfCCT(528-795) K663A 10 µM
PfCCT(528-795) K663A Ø PPase,ChoP 10 µM
PfCCT(528-795) K663A Ø CTP 10 µM
Absorbance (360 nm) (AU)
w/o PfCCT
PfCCT addition
t (s)
c
CTP titration
v0 (s-1)
cCTP (mM)
d
ChoP titration
v0 (s-1)
cChoP (mM)
e
Time (min)
Power (µcal·s-1)
kcal·mol-1
[CTP]/[PfCCT(528-795) K663A]
f
Time (min)
Power (µcal·s-1)
kcal·mol-1
[CDPCho]/[PfCCT(528-795) K663A]

Supplementary Figure S4. Enzymatic and ligand binding characterization of *Pf*CCT$_{(528\text{-}795)}$ K663A mutant (**a**) Schematic representation of the catalytic mechanism of *Pf*CCT$_{(528\text{-}795)}$ and *Pf*CCT$_{(528\text{-}795)}$ K663A mutant. In case of the *Pf*CCT$_{(528\text{-}795)}$ (top) the CTP+ChoP→CDPCho+PP$_i$ conversion (cytidylyltransferase activity) is followed by the steps catalyzed by the auxiliary enzymes, pyrophosphatase (PPase) and purine nucleoside phosphorylase (PNP), yielding the 7-methyl-6-thioguanine (MES) product which produces the absorbance change signal (Absorbance 360 nm). *Pf*CCT$_{(528\text{-}795)}$ K663A (below) presumably catalyzes CTP phosphohydrolase reaction where the MESG/MES interconversion revealed the P$_i$ produced in the CTP→CDP+P$_i$ reaction. (**b**) Continuous photometric assay of measuring CCT enzymatic activity. The figure presents real datasets measured at 1 mM CTP and 5 mM ChoP concentration for *Pf*CCT$_{(528\text{-}795)}$ (dark grey) and *Pf*CCT$_{(528\text{-}795)}$ K663A (orange). A control experiment with 5 mM ChoP and without CTP (light grey) didn't show any activity upon *Pf*CCT$_{(528\text{-}795)}$ K663A addition. On the contrary, when the experiment was done with 1 mM CTP but without the other substrate ChoP and the auxiliary enzyme PPase, an activity was detected upon *Pf*CCT$_{(528\text{-}795)}$ K663A addition (yellow). This activity could be assigned to a phosphohydrolase activity of the mutant enzyme. The corresponding timescale is indicated on the X axis. (**c**) CTP titration of the activity of *Pf*CCT$_{(528\text{-}795)}$ K663A at a fixed ChoP concentration of 5 mM. (**d**) ChoP titration *Pf*CCT$_{(528\text{-}795)}$ K663A at a fixed CTP concentration of 1 mM. Note that the activity of *Pf*CCT$_{(528\text{-}795)}$ K663A does not display saturation with increasing concentration of ChoP. Mean and SD of three replicates shown. (**e**) Equilibrium CTP binding to *Pf*CCT$_{(528\text{-}795)}$ K663A followed by ITC. Titration of 288 µM *Pf*CCT$_{(528\text{-}795)}$ K663A with 7.27 mM CTP performed at 20 °C is shown. (**f**) Equilibrium CDPCho binding to *Pf*CCT$_{(528\text{-}795)}$ K663A followed by ITC. Titration of 167 µM *Pf*CCT$_{(528\text{-}795)}$ K663A with 5.8 mM CDPCho performed at 20 °C is shown. Values of the measured kinetic and thermodynamic parameters and their errors are denoted in Table 1 and Table 2, respectively.

Figure S5

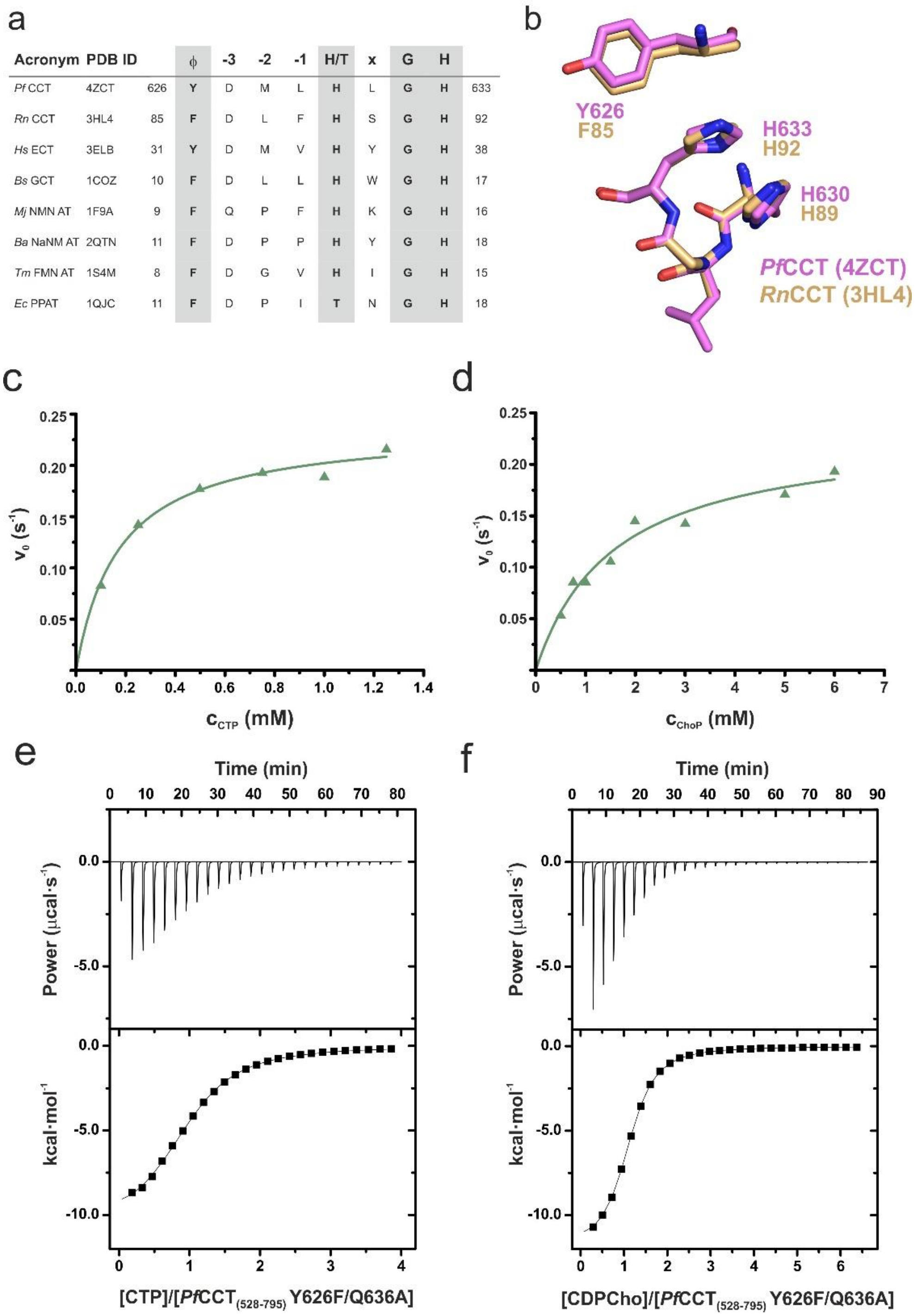

Supplementary Figure S5. Kinetic and ligand binding characterization of *Pf*CCT(528-795) Y626F/Q636A mutant. (**a**) Sequence alignment reveals the conservation of an aromatic residue (ϕ) preceding the HxGH motif conserved in the Rossmann fold nucleotidyltransferase enzyme family. The selected representatives of the enzyme family includes *Plasmodium falciparum* CCT (*Pf*CCT), *Rattus norvegicus* CCT (*Rn*CCT)[2], *Homo sapiens* CTP:ethanolamine

cytidylyltransferase (*Hs*ECT)[3], *Bacillus subtilis* GCT:glycerol-3-phosphate cytidylyltransferase (*Bs*GCT)[4], *Methanococcus jannashii* nicotinamide mononucleotide adenylyltransferase (*Mj* NMN AT)[5], *Bacillus anthracis* nicotinic acid mononucleotide adenylyltransferase (*Ba* NaMN AT)[6], *Thermotoga maritima* FAD synthetase (*Tm* FMN AT)[7], *Escherichia coli* Phosphopantetheine adenylyltransferase (*Ec* PPAT)[8]. (**b**) Superimposition of *Pf*CCT (PDB: 4ZCT) (violet) and *Rn*CCT (PDB: 3HL4) (light orange) catalytically important histidine residues (alignment is shown in panel a). Note the edge-on contact of Y626 (F85) and H633 (H92). (**c**) CTP kinetic titration of *Pf*$CCT_{(528\text{-}795)}$ Y626F/Q636A at a fixed ChoP concentration of 5 mM. (**d**) ChoP titration of the activity of *Pf*$CCT_{(528\text{-}795)}$ Y626F/Q636A at a fixed CTP concentration of 1 mM. For **c**) and **d**) Kinetic data are fitted with the Michaelis–Menten equation. (**e**) Equilibrium CTP binding to *Pf*$CCT_{(528\text{-}795)}$ Y626F/Q636A followed by ITC. Titration of 340 µM *Pf*$CCT_{(528\text{-}795)}$ Y626F/Q636A with 6.34 mM CTP performed at 20 °C is shown. (**f**) Equilibrium CDPCho binding to *Pf*$CCT_{(528\text{-}795)}$ Y626F/Q636A followed by ITC. Titration of 235 µM *Pf*$CCT_{(528\text{-}795)}$ Y626F/Q636A with 7.18 mM CDPCho performed at 20 °C is shown. Values of the measured kinetic and thermodynamic parameters and their errors are denoted in Table 1 and Table 2, respectively.

Figure S6

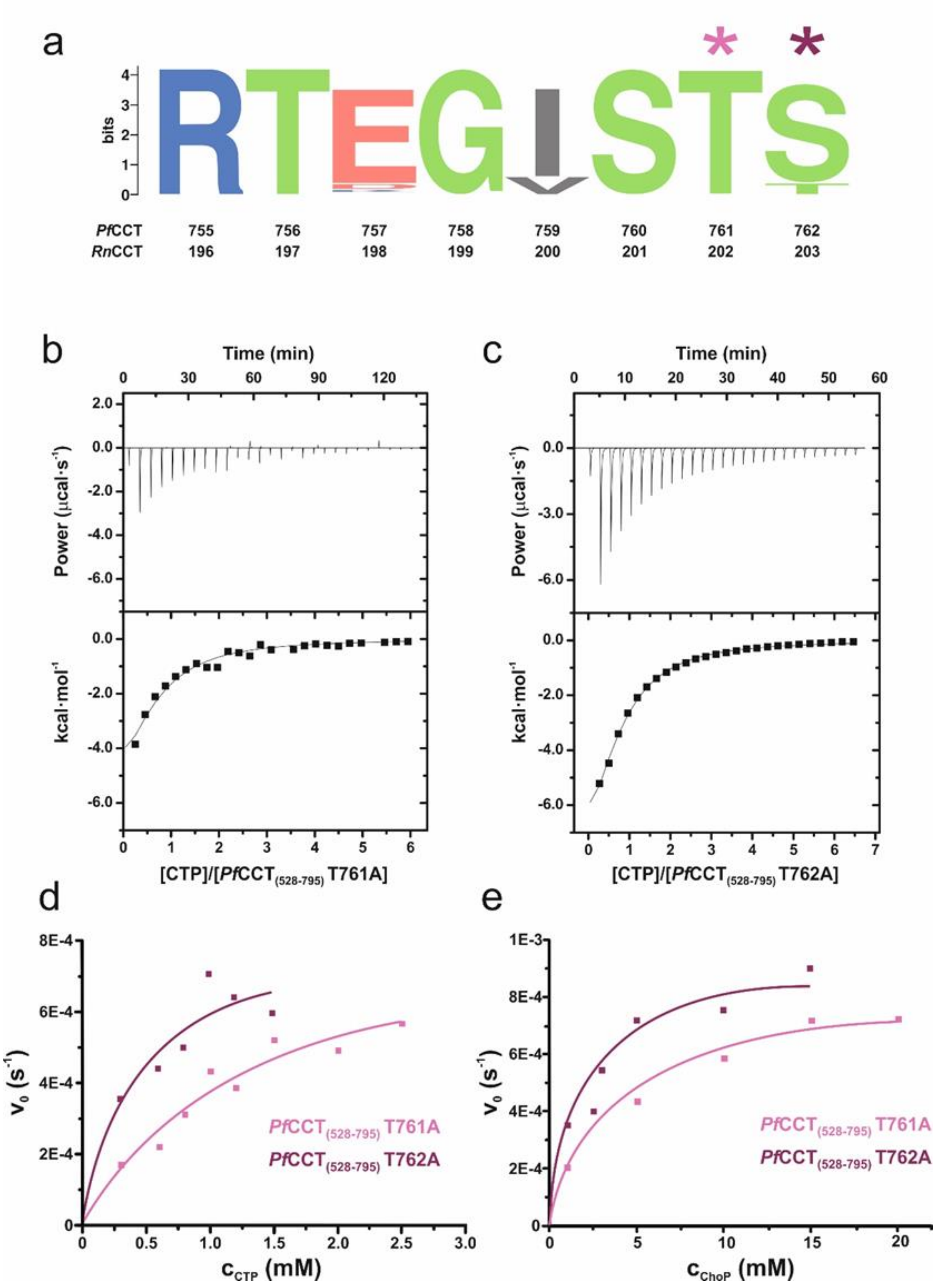

a
bits
R T E G I S T S
PfCCT 755 756 757 758 759 760 761 762
RnCCT 196 197 198 199 200 201 202 203
b
Time (min)
Power (µcal·s-1)
kcal·mol-1
[CTP]/[PfCCT(528-795) T761A]
c
Time (min)
Power (µcal·s-1)
kcal·mol-1
[CTP]/[PfCCT(528-795) T762A]
d
v0 (s-1)
cCTP (mM)
PfCCT(528-795) T761A
PfCCT(528-795) T762A
e
v0 (s-1)
cChoP (mM)
PfCCT(528-795) T761A
PfCCT(528-795) T762A

Supplementary Figure S6. Kinetic and ligand binding characterization of *Pf*CCT$_{(528-795)}$ T761A and T762A mutants. (**a**) Sequence alignment demonstrates high conservation of the RTEGIST(S/T) signature sequence in CCT enzymes. A non-redundant protein-protein BLAST run was performed with the rat CCT (UniProt ID: P19836) as input sequence at the GenomeNet portal (http://www.genome.jp). A subsequent multiple sequence alignment was performed on a refined hit list including only those hit sequences that were unambiguously annotated as CCT (231 items). The sequence alignment was transferred to WebLogo[9] using default settings. The mutated residues T761 and T762 are indicated by pink and purple stars, respectively. (**b**) Equilibrium CTP binding to *Pf*CCT$_{(528-795)}$ T761A followed by ITC. Titration of 323 µM *Pf*CCT$_{(528-795)}$ T761A with 8.94 mM CTP performed at 20 °C is shown. (**c**) Equilibrium CTP binding to *Pf*CCT$_{(528-795)}$ T762A followed by ITC. Titration of 297 µM *Pf*CCT$_{(528-795)}$ T762A with 8.91 mM CTP performed at 20 °C is shown. (**d**) CTP titration of the *Pf*CCT$_{(528-795)}$ T761A activity (pink) and *Pf*CCT$_{(528-795)}$ T762A activity (purple) at a fixed ChoP concentration of 5 mM. (**e**) ChoP titration of the *Pf*CCT$_{(528-795)}$ T761A activity (pink) and *Pf*CCT$_{(528-795)}$ T762A activity (purple) at a fixed CTP concentration of 1 mM. Kinetic titration data of *Pf*CCT$_{(528-795)}$ T761A and *Pf*CCT$_{(528-795)}$ T762A shown in **d**) and **e**) are fitted with the Michaelis–Menten equation. Values of the measured kinetic and thermodynamic parameters and their errors are given in Table 1 and Table 2, respectively.

Table S1. Root Mean Square Deviations (RMSDs) between *Pf*CCT structures. The RMSD values in Å obtained for all atoms were calculated using PyMol[10]. The number of atoms for which RMSDs were calculated is given in parenthesis.

	free PfCCT	***CDPCho-PfCCT***	***CMP-PfCCT***	***ChoP-PfCCT***
CDPCho-PfCCT	2.20 (1023)	×		
CMP-PfCCT	0.84 (978)	0.85 (1018)	×	
ChoP-PfCCT	1.14 (988)	1.16 (1024)	0.66 (1005)	×
Cho-PfCCT	1.34 (1002)	1.13 (1002)	1.07 (997)	1.04 (1002)

Table S2. Interaction distances of *Pf*CCT and its ligands, CDPCho, CMP and ChoP.

Ligand	*Pf*CCT atom	Ligand atom	Distance
CDPCho	T756 O	Cytosine-N4	2.9 Å
	T756 N	Cytosine-N3	3.3 Å
	R755 N	Ribose 2' OH	3.9 Å
	R755 N	Cytosine-N1	4.1 Å
	H633 N-ε2	α-Phosphate O1	3.7 Å
	D710 O-δ2	Ribose 2' OH	2.3 Å
	Q636 N-ε2	Ribose 3' OH	3.2 Å
	H709 N-ε2	β-Phosphate O2	2.9 Å
	Y626 N	α-Phosphate O1	2.8 Å
	V625 N	α-Phosphate O3	3.2 Å
	K663 N-ξ	α-Phosphate O2	3.0 Å
	K663 N-ξ	β-Phosphate O1	2.9 Å
	Y714 O-η	β-Phosphate O1	2.8 Å
CMP	T756 O	Cytosine-N4	2.7 Å
	T756 N	Cytosine-N3	3.2 Å
	R755 N	Ribose 2' OH	3.4 Å
	R755 N	Cytosine-N1	4.0 Å
	D710 O-δ2	Ribose 2' OH	2.5 Å
	Q636 N-ε2	Ribose 3' OH	3.5 Å
	H633 N-ε2	Phosphate O1	3.0 Å
	Y626 N	Phosphate O1	2.8 Å
	V625 N	Phosphate O3	2.7 Å
ChoP	H709 N-ε2	Phosphate O2	2.8 Å
	V625 N	Phosphate O3	3.0 Å
	K663 N-ξ	Phosphate O1	2.9 Å

Note that R755 is found in different conformations within the various liganded states and thus in some cases its distance from ligand moieties exceeds 3.5 Å.

Table S3. Primer sequences used for mutagenesis

Protein construct	forward primer	reverse primer
*Pf*CCT$_{(581-775)}$	CATATGGCCGTTCCGGACG	GGATCCTTAGTAATCTTCG
*Pf*CCT$_{(528-795)}$ Y626F/Q636A	TGGTGTTTTCGACATGCTGCAC CTGGG	GCATGTCGAAAACACCATCGGCGTA AATC
	ACATGAAAGCACTGGAACAAG CCAAAAAACTG	GTTCCAGTGCTTTCATGTGACCCAGG TGC
*Pf*CCT$_{(528-795)}$ T761A	GTACGGAAGGCGTGTCTGCGA CGGACCTGATCG	CGATCAGGTCCGTCGCAGACA CGCCTTCCGTAC
*Pf*CCT$_{(528-795)}$ T762A	CGGAAGGCGTGTCTACGGCGG ACCTGATCGTCCG	CGGACGATCAGGTCCGCCGTAGACA CGCCTTCCG
*Pf*CCT$_{(528-795)}$ K663A	CAACGAAACCAAACTGTTTGC AGGCCAGGTTGTCC	GGACAACCTGGCCTGCAAACAGTTT GGTTTCGTTG

References

1 Winn, M. D. *et al.* Overview of the CCP4 suite and current developments. *Acta Crystallogr. D Biol. Crystallogr.* **67**, 235-242, (2011).

2 Lee, J., Johnson, J., Ding, Z., Paetzel, M. & Cornell, R. B. Crystal structure of a mammalian CTP: phosphocholine cytidylyltransferase catalytic domain reveals novel active site residues within a highly conserved nucleotidyltransferase fold. *J. Biol. Chem.* **284**, 33535-33548, (2009).

3 Tian, S. *et al.* Human CTP:phosphoethanolamine cytidylyltransferase: enzymatic properties and unequal catalytic roles of CTP-binding motifs in two cytidylyltransferase domains. *Biochem. Biophys. Res. Commun.* **449**, 26-31, (2014).

4 Weber, C. H., Park, Y. S., Sanker, S., Kent, C. & Ludwig, M. L. A prototypical cytidylyltransferase: CTP:glycerol-3-phosphate cytidylyltransferase from bacillus subtilis. *Structure* **7**, 1113-1124, (1999).

5 D'Angelo, I. *et al.* Structure of nicotinamide mononucleotide adenylyltransferase: a key enzyme in NAD(+) biosynthesis. *Structure* **8**, 993-1004, (2000).

6 Sershon, V. C., Santarsiero, B. D. & Mesecar, A. D. Kinetic and X-ray structural evidence for negative cooperativity in substrate binding to nicotinate mononucleotide adenylyltransferase (NMAT) from Bacillus anthracis. *J. Mol. Biol.* **385**, 867-888, (2009).

7 Wang, W., Kim, R., Yokota, H. & Kim, S. H. Crystal structure of flavin binding to FAD synthetase of Thermotoga maritima. *Proteins* **58**, 246-248, (2005).

8 Izard, T. The crystal structures of phosphopantetheine adenylyltransferase with bound substrates reveal the enzyme's catalytic mechanism. *J. Mol. Biol.* **315**, 487-495, (2002).

9 Crooks, G. E., Hon, G., Chandonia, J. M. & Brenner, S. E. WebLogo: a sequence logo generator. *Genome Res.* **14**, 1188-1190, (2004).

10 DeLano, W. L. The PyMOL Molecular Graphics System. http://www.pymol.org, DeLano Scientific, San Carlos, CA, USA, (2002).
